# Evolution, expression and functional analysis of cultivated allotetraploid cotton *DIR* genes

**DOI:** 10.1186/s12870-021-02859-0

**Published:** 2021-02-10

**Authors:** Zhengwen Liu, Xingfen Wang, Zhengwen Sun, Yan Zhang, Chengsheng Meng, Bin Chen, Guoning Wang, Huifeng Ke, Jinhua Wu, Yuanyuan Yan, Liqiang Wu, Zhikun Li, Jun Yang, Guiyin Zhang, Zhiying Ma

**Affiliations:** State Key Laboratory of North China Crop Improvement and Regulation, North China Key Laboratory for Crop Germplasm Resources of Education Ministry, Hebei Agricultural University, Baoding, 071001 China

**Keywords:** Cotton, Dirigent proteins, Evolution, RNA-seq, Verticillium wilt, Fiber development, Overexpression

## Abstract

**Background:**

Dirigent (DIR) proteins mediate regioselectivity and stereoselectivity during lignan biosynthesis and are also involved in lignin, gossypol and pterocarpan biosynthesis. This gene family plays a vital role in enhancing stress resistance and in secondary cell-wall development, but systematical understanding is lacking in cotton.

**Results:**

In this study, 107 *GbDIRs* and 107 *GhDIRs* were identified in *Gossypium barbadense* and *Gossypium hirsutum*, respectively. Most of these genes have a classical gene structure without intron and encode proteins containing a signal peptide. Phylogenetic analysis showed that cotton *DIR* genes were classified into four distinct subfamilies (a, b/d, e, and f). Of these groups, DIR-a and DIR-e were evolutionarily conserved, and segmental and tandem duplications contributed equally to their formation. In contrast, DIR-b/d mainly expanded by recent tandem duplications, accompanying with a number of gene clusters. With the rapid evolution, DIR-b/d-III was a *Gossypium*-specific clade involved in atropselective synthesis of gossypol. RNA-seq data highlighted *GhDIR*s in response to *Verticillium dahliae* infection and suggested that *DIR* gene family could confer Verticillium wilt resistance. We also identified candidate *DIR* genes related to fiber development in *G. barbadense* and *G. hirsutum* and revealed their differential expression. To further determine the involvement of *DIR* genes in fiber development, we overexpressed a fiber length-related gene *GbDIR78* in *Arabidopsis* and validated its function in trichomes and hypocotyls.

**Conclusions:**

These findings contribute novel insights towards the evolution of *DIR* gene family and provide valuable information for further understanding the roles of *DIR* genes in cotton fiber development as well as in stress responses.

**Supplementary Information:**

The online version contains supplementary material available at 10.1186/s12870-021-02859-0.

## Background

The genus *Gossypium* contains at least 46 diploids and five well-established allotetraploids. Two allotetraploid species, *G. hirsutum* and *G. barbadense*, have been cultivated worldwide, which originated from one hybridization event between A-genome (resembling *G. arboreum* or *G. herbaceum*) and D-genome (resembling *G. raimondii*) progenitors around 1–2 million years ago (MYA) [1, 2]. *G. hirsutum* accounts for more than 90% of the global cotton production because of its high yield, whereas *G. barbadense* is characterized by its superior fiber properties. Cotton faces biotic and abiotic stresses during its growth; in addition, improved fiber quality became an urgent need to address the challenges of synthetic fiber industry. The most economic and effective strategy is to develop genetically modified cultivars that not only enhance resistance against environmental stresses but also improve fiber quality. Thus, it is important to mine candidate genes and explain their mechanisms.

Dirigent (from Latin *dirigere*, to guide or align) protein was first isolated from *Forsythia intermedia* and mediated regioselective and stereoselective coupling of two coniferyl alcohol-derived radicals during (+)-pinoresinol biosynthesis [3]. After that, (+)-pinoresinol-forming DIR proteins have been cloned from several seed-bearing plants, such as *Thuja plicata* [4], *Schisandra chinensis* [5], *Pisum sativum* [6], *Linum usitatissimum* [7] and *Glycine max* [8]. Additionally, (−)-pinoresinol-forming DIR proteins from *Arabidopsis thaliana* [5] and *Linum usitatissimum* [7] were also isolated and characterized. Lignin also derives from monolignol polymerization but is optically inactive. Although molecular mechanisms remain obscure, DIR proteins have been involved in lignin biosynthesis [9–11]. Besides the monolignol metabolism, a few cotton DIR proteins were proposed to mediate the formation of (+)-gossypol by atropselective coupling of hemigossypol radicals [12, 13]. DIR proteins typically lack a catalytically active center, but some members in leguminous plants, with isoflavanol dehydratase activity, can produce pterocarpans [14].

Having the various biochemical functions, DIR proteins play important roles in stress responses, especially in plant defense against pathogens. The expression of *PsDRR206* in pea pod tissue was induced by *Fusarium solani* infection, and its metabolite functioned as phytoalexin [6]. Overexpression of *GmDIR22* in soybean can increase total lignan accumulation and enhance plant resistance to *Phytophthora sojae* [8]. Pepper plants with the silencing of *CaDIR7* are more susceptible to *Phytophthora capsici*, NaCl and mannitol stresses [15]. Cotton plants with the overexpression of *GhDIR1* showed an increased lignin content and displayed more resistance to *Verticillium dahliae* [11]. Besides defense responses, DIR proteins also presented other kinds of physiological functions such as Casparian strip formation [10] and pod dehiscence [16].

Ralph and colleagues performed a phylogenetic analysis of 150 DIR proteins from the seed plant division and suggested the presence of six distinct DIR subfamilies, DIR-a and five DIR-like subfamilies (b/d, c, e, f and g) [17]. After that, *DIR* gene family has been systematically studied in several vascular plants, and some stress-induced genes [15, 18–20] or SCW-related genes [21, 22] were identified. Understanding the functions of *DIR* genes would be a practicable approach to enhance stress resistance of cotton and to improve fiber properties, but no comprehensive understanding has been provided in cotton until now. There are only a few *V. dahliae*-responsive *DIR* genes in literatures, and fiber development-related *DIR* genes remain to be identified. Recently, a series of *Gossypium* whole-genome sequences has been released, which makes it possible to analyze gene family at a genome-wide level in cotton. In the present study, we firstly identified the *DIR* gene family members in cultivated allotetraploid cotton, *G. barbadense* and *G. hirsutum*, and analyzed their evolution. Their expression patterns in response to *V. dahliae* infection and during cotton fiber development were systematically investigated. Furthermore, the functional analysis of *GbDIR78* in *Arabidopsis* revealed its role in cell elongation. Our findings will further the understanding of this elusive gene family and provide candidate *DIR* genes for both defense response and fiber development.

## Results

### Identification, characterization and phylogenetic analysis of *DIR* genes

**Fig. 1:**
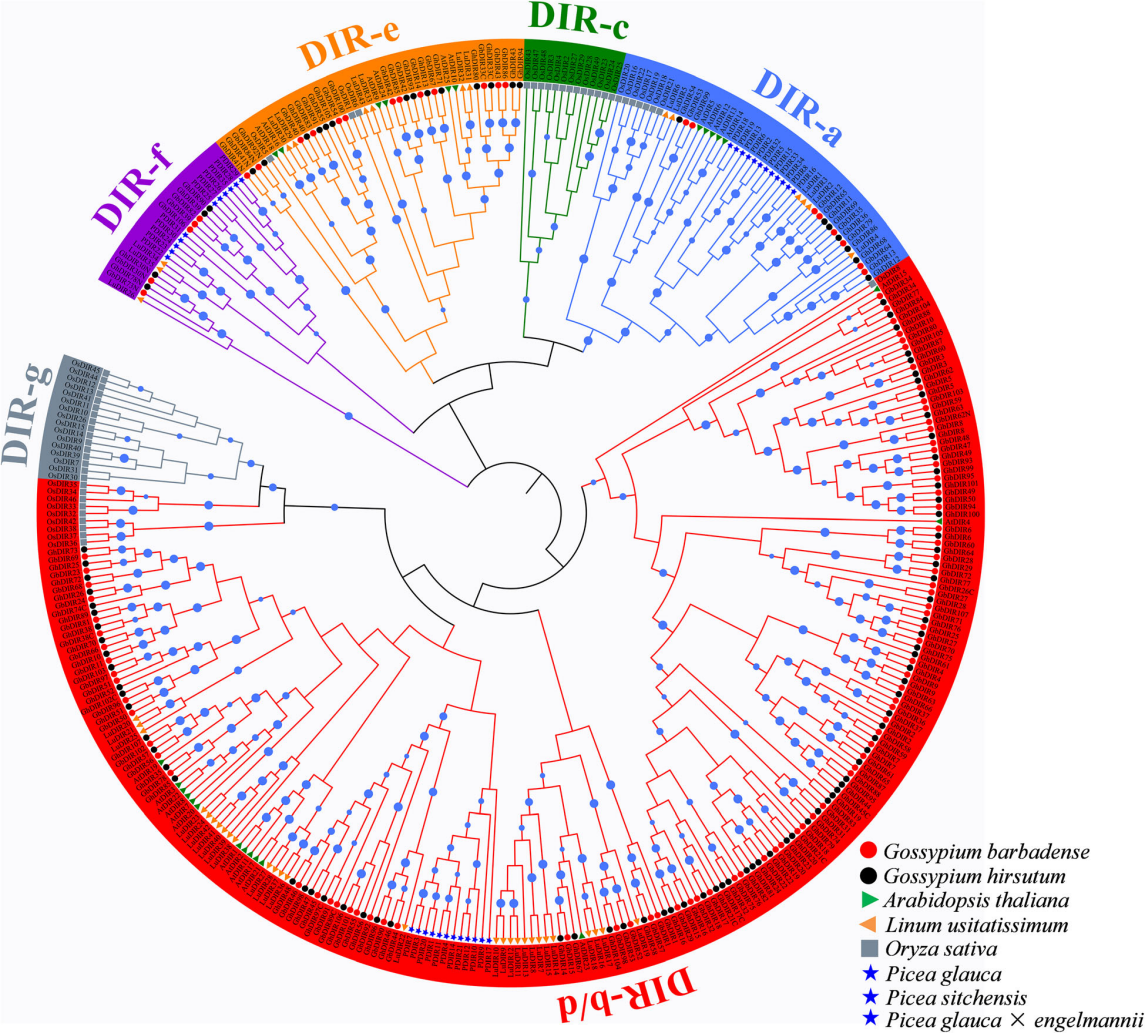
Phylogenetic relationships of *DIR* genes from *G. barbadense*, *G. hirsutum*, *A. thaliana*, *L. usitatissimum*, *O. sativa* and *Picea*. The phylogenetic tree was constructed by MEGA 7.0 using the neighbor-joining (NJ) method with 1000 bootstrap replicates. Bootstrap values > 50% are shown by blue solid circles on the branches, and their sizes reflect the reliability

We identified 107 *GbDIR* genes (*GbDIR1*-*GbDIR107*) in *G. barbadense* and 107 *GhDIR* genes (*GhDIR1*-*GhDIR107*) in *G. hirsutum* (Table 1; Additional file 8: Table S1 and S2). To understand the evolutionary relationships, a neighbor-joining (NJ) phylogenetic tree was constructed using the coding sequences of 107 *GbDIRs*, 107 *GhDIRs*, 25 *AtDIRs* (*Arabidopsis thaliana*), 44 *LuDIRs* (*Linum usitatissimum*), 49 *OsDIRs* (*Oryza sativa*) and 35 *PDIRs* (*Picea* spp.) (Additional file 8: Table S3). As shown in Fig. 1, *DIR* genes were clearly divided into six subfamilies, designated as DIR-a, DIR-b/d, DIR-c, DIR-e, DIR-f and DIR-g. The topological structure is essentially in agreement with previous reports [17, 19, 21, 23]. Among these subfamilies, DIR-a consisted of 8 *GbDIRs*, 7 *GhDIRs*, 5 *AtDIRs*, 6 *LuDIRs*, 7 *OsDIRs* and 12 *PDIRs*, indicating a highly-conserved ancient clade. The largest group DIR-b/d consisted of 81 *GbDIRs*, 82 *GhDIRs*, 14 *AtDIRs*, 28 *LuDIRs*, 10 *OsDIRs* and 12 *PDIRs*, showing that DIR-b/d has expanded considerably in allotetraploid cotton. In contrast, DIR-f only included 5 *GbDIRs*, 4 *GhDIRs*, 3*LuDIRs* and 11 *PDIRs*, probably due to gene losses in *Arabidopsis thaliana* and *Oryza sativa*. DIR-e contained 13 *GbDIRs*, 14 *GhDIRs*, 6 *AtDIRs*, 6 *LuDIRs*, 3 *OsDIRs* but no *PDIRs*, suggesting a possible angiosperm-specific clade. DIR-c and DIR-g might be unique to monocotyledons, for they only contained *OsDIRs*. To validate the phylogenetic relationships, we reconstructed two phylogenetic trees using DIR protein sequences of *G. barbadense* and *G. hirsutum* (Additional file 1: Figure S1). The evolutionary relationships were highly consistent with that in Fig. 1. Furthermore, these DIR protein sequences were submitted to MEME to discover conserved motifs. As shown in Additional file 1: Figure S1, the adjacent clades carried similar motifs.

**Table 1:** Summary of *DIR* genes in *G. barbadense* and *G. hirsutum*

Species	Class	No. of genes	Mapped chromosomes	SD^a^	TD^a^	MW^b^	pI^b^	N-gly^b^
*G. barbadense*	a	8	6	5	4	21.0	9.2	3.5
	e	13	7	4	6	27.5	5.1	0.0
	f	5	4	0	0	20.1	8.7	3.0
	b/d-I	29	16	4	10	21.1	9.7	3.0
	b/d-II	28	9	0	2	20.5	7.9	3.0
	b/d-III	24	7	0	9	19.9	7.0	5.0
	Total	107	23	13	31	20.8	8.5	3.0
*G. hirsutum*	a	7	5	4	2	20.9	9.1	4.0
	e	14	8	6	5	27.7	5.1	0.0
	f	4	4	0	0	19.9	8.8	3.0
	b/d-I	31	18	8	12	21.0	9.7	3.0
	b/d-II	23	9	0	6	20.9	8.4	3.0
	b/d-III	28	8	0	16	19.2	6.8	5.0
	Total	107	24	18	41	20.8	8.4	3.0

^a^ In each class, *DIR* genes involved in segmental duplication (SD) and tandem duplication (TD) were counted. ^b^ In each class, the properties of DIR proteins including molecular weight (MW), isoelectric point (pI), and the number of putative N-glycosylation sites (N-gly) were investigated, and the median values were displayed

Most cotton *DIR* genes (especially members in DIR-a, DIR-e and DIR-f) held a classical gene structure without intron (Additional file 1: Figure S1). Notably, the DIR-b/d clade showed variable gene structures, indicating lower selection constraints. About 77% of *GbDIRs* and 82% of *GhDIRs* encoded proteins containing a signal peptide (Additional file 8: Table S1 and S2). As expected, subcellular localization prediction showed that they were mainly located in the extracellular space. In spite of having a signal peptide, several DIR proteins belonging to DIR-b/d were directed to the vacuole. In addition, DIR proteins located in the nucleus or the peroxisome were observed; the unexpected localization might signify novel functions. We also scanned cotton DIR protein sequences for N-glycosylation sites to explore their solubility and stability. Interestingly, DIR-e genes had much fewer N-glycosylation sites (Table 1), displaying the evolution and divergence of cotton *DIR* gene family.

### Chromosomal distribution, duplication and evolution

To determine the chromosomal distribution of cotton *DIR* genes, we marked their physical locations on chromosomes based on their annotation information (Additional file 2: Figure S2). All the 107 *GhDIRs* were mapped to 24 of the total 26 chromosomes (i.e. except At06 and Dt06). The gene number varied from 1 to 15 across these chromosomes. Specifically, *GhDIRs* belonging to DIR-b/d were enriched on chromosomes At01, At04, At11, Dt01, Dt04 and Dt11, while DIR-e genes were mainly located on At10 and Dt10. Gene clusters were frequently observed, indicating a number of tandem duplication events. Because of the short divergence time, *GbDIRs* and *GhDIRs* were extremely similar in chromosomal distribution. *GbDIRs* were located on chromosomes except Dt02, At06 and Dt06, and the gene number ranged from 1 to 13. Six genes (*GbDIR29* and *GbDIR103*-*GbDIR107*) were not mapped because of the incomplete location information.

**Fig. 2:**
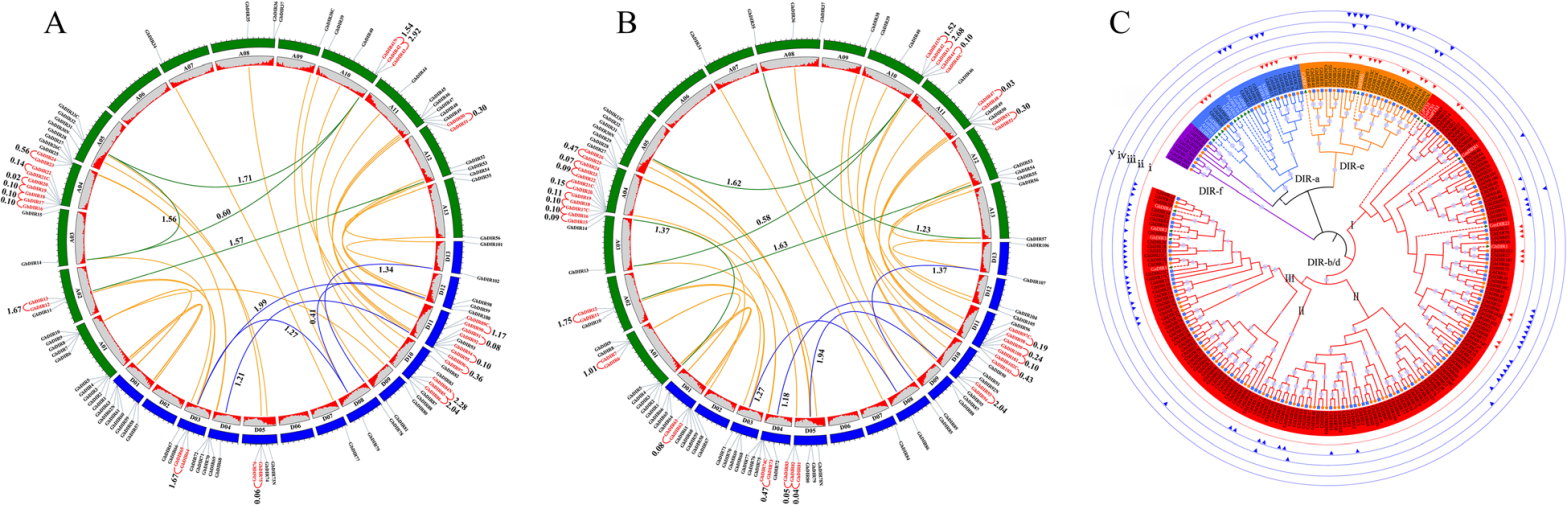
Duplication events among *DIR* genes. **a** Segmental and tandem duplications in *G. barbadense*. Green and blue lines indicate segmental duplications within At- and Dt-subgenome, respectively. Red lines indicate tandem duplications. Ks values are displayed next to the lines. Orange lines link homologous genes between At- and Dt-subgenome. **b** Segmental and tandem duplications in *G. hirsutum*. **c** The expansion of cotton *DIR* gene family. The phylogenetic tree consists of 107 *GbDIRs*, 107 *GhDIRs* and 23 reported *DIRs* (in a white font). Track i marks segmental duplications, while tracks ii, iii, iv and v mark tandem duplications with Ks ranges of 0.0–0.2, 0.2–0.6, 0.6–1.9 and 1.9–3.0, respectively

To analyze the expansion of cotton *DIR* gene family, we identified segmental and tandem duplications in *G. barbadense* and *G. hirsutum*. The numbers of segmentally duplicated *DIR* genes and tandemly duplicated *DIR* genes were 13 and 31, respectively, in *G. barbadense* and 18 and 41 in *G. hirsutum* (Table 1; Fig. 2a and b; Additional file 8: Table S4 and S5). Obviously, tandem duplication as the major impetus drove the expansion of cotton *DIR* gene family, corresponding to the above-mentioned gene clusters. Ks (substitution per synonymous site) values can be used to estimate the occurrence time of gene duplications and then to identify whole-genome duplication (WGD) events. Ks ranges of 0.4–0.6 (corresponding to *Gossypium*-specific WGD around 16.6 MYA) and 1.5–1.9 (corresponding to the paleo-hexaploidization event shared by eudicots around 130.8 MYA) were observed in *G. raimondii* [24]. Moreover, Ks peak of 0.03 accounts for the divergence between A-genome and D-genome progenitors [25]. Here we also calculated the Ks values of gene duplications to analyze their occurrence time; the LPB method was selected because its results were just in line with expectations (Additional file 8: Table S4 and S5). As shown in Fig. 2, Ks values of *GbDIR14*/*GbDIR41*, *GbDIR79*/*GbDIR99* and *GhDIR13*/*GhDIR41* were 0.60, 0.41 and 0.58, respectively, indicating that these three segmental duplications might be generated from *Gossypium*-specific polyploidization. The other segmental duplications were inferred to arise from the paleo-hexaploidization event, for their Ks values were in or near 1.5–1.9. Interestingly, segmentally duplicated *DIR* genes were mainly retained from the ancestral WGD event rather than the recent one, suggesting some evolutionarily conserved genes. Ks values of the tandem duplications ranged from 0.02 to 2.92 in *G. barbadense* and from 0.03 to 2.68 in *G. hirsutum*. Most of the tandem duplications had Ks value less than 0.5, showing a recent expansion of cotton *DIR* gene family. The lacking Ks values of 0.5–1.0 might mean gene losses and/or translocations due to chromosome re-packaging processes following polyploidization. Besides, some tandem duplications with Ks > 1.0 were observed, suggesting that these genes were quite stable.

To further reveal evolutionary history of cotton *DIR* gene family, we integrated the duplication events with a phylogenetic tree containing 107 *GbDIRs*, 107 *GhDIRs* and 23 reported *DIRs* (Fig. 2c; Additional file 8: Table S6). Track i marked segmental duplications, while tracks ii, iii, iv and v (with artificially-created Ks ranges of 0.0–0.2, 0.2–0.6, 0.6–1.9 and 1.9–3.0, respectively) labeled tandem duplications. The values 0.6 and 1.9 referred to above-mentioned 0.4–0.6 and 1.5–1.9, respectively, and a cluster of Ks values around 0.1 set up the Ks range of 0.0–0.2 (Additional file 8: Table S4 and S5). The DIR-a clade, mainly involved in lignan biosynthesis, was basically established before the *Gossypium*-specific WGD event (Fig. 2c). Similarly, DIR-e was also a relatively ancient clade because of the lack of recent duplications. Clearly, DIR-b/d has experienced rapid expansion due to recent tandem duplications; we believed DIR-b/d-I, −II and -III arose in turn in evolution. Among them, DIR-b/d-I covered tandem duplications and segmental duplications, forming a transition clade. DIR-b/d-II and the right half of DIR-b/d-III, lacking traces of duplication events, might have undergone large-scale gene losses and/or translocations. The left half of DIR-b/d-III, involved in the atropselective synthesis of gossypol, emerged recently due to tandem duplications. In addition, DIR-f was obviously inactive in cotton evolution (Table 1).

The Ka/Ks ratio is a measure of selective pressure on proteins, and Ka/Ks > 1, = 1 and < 1 indicate positive selection (or molecular adaptation), neutral evolution, and purifying selection (or selective constraints), respectively. Here we calculated the Ka/Ks ratio for all the duplicated *DIR* gene pairs (Additional file 8: Table S4 and S5). Interestingly, those three gene pairs generated from *Gossypium*-specific WGD (i.e. *GbDIR14*/*GbDIR41*, *GbDIR79*/*GbDIR99* and *GhDIR13*/*GhDIR41*) showed Ka/Ks ratios > 1, implying that positive selection might contribute to their surviving from gene losses and/or translocations. Ka/Ks ratios of the other segmentally duplicated *DIR* gene pairs were fairly low (0.14–0.35 in *G. barbadense* and 0.14–0.34 in *G. hirsutum*), suggesting the conserved functions due to purifying selection (Additional file 3: Figure S3a and S3b). Moreover, tandemly duplicated gene pairs belonging to DIR-a and DIR-e also showed very small Ka/Ks values (0.11–0.38 in *G. barbadense* and 0.13–0.39 in *G. hirsutum*), further showing the conservation of these two clades. In contrast, gene pairs in the DIR-b/d-III clade held larger Ka/Ks values (0.51–1.74 *in G. barbadense* and 0.53–1.04 *in G. hirsutum*), indicating weaker selective constraints (Additional file 3: Figure S3c and S3d). The moderate selection pressure might result in the rapid expansion of DIR-b/d-III.

### Evolutionary history of cotton *DIR* gene family

**Fig. 3:**
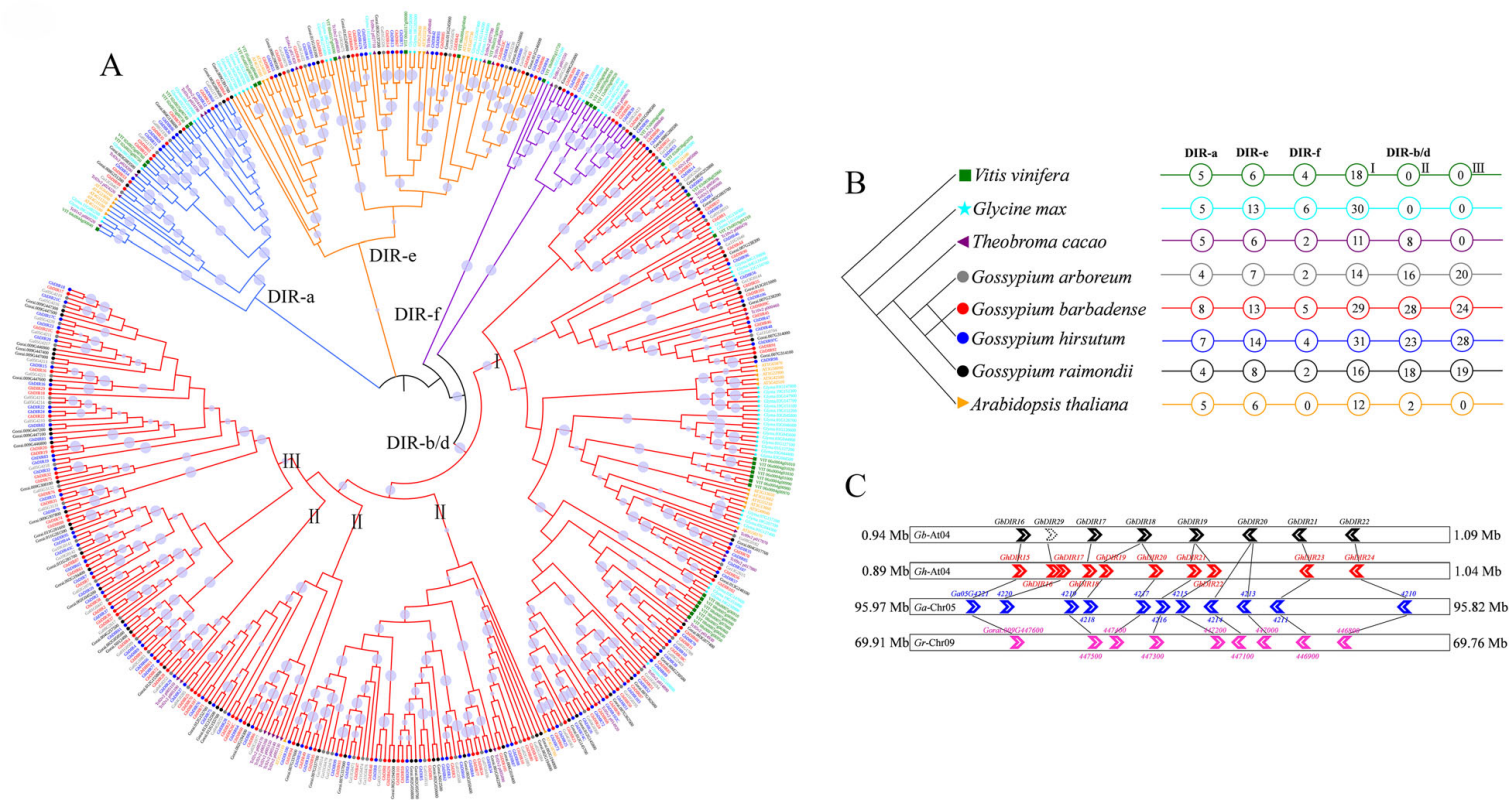
Evolutionary history of cotton *DIR* gene family. **a** Phylogenetic relationships of *DIR* genes from *V. vinifera*, *G. max*, *A. thaliana*, *T. cacao*, *G. arboreum*, *G. raimondii*, *G. barbadense* and *G. hirsutum*. **b** *DIR* genes were assigned to distinct clades. The figures in circles stand for the number of genes. **c** The collinearity among multiple *Gossypium* genomes. Lines between chromosomes show orthologous genes

To further verify the evolution of cotton *DIR* gene family, we constructed a phylogenetic tree consisting of 488 *DIR* genes from eight dicotyledonous species sharing a common paleo-hexaploid ancestor (Fig. 3a; Additional file 8: Table S7). After the paleo-hexaploidization event, *Arabidopsis thaliana* and *Glycine max* have severally undergone two rounds of WGD events, while no WGDs have been identified in *Vitis vinifera* and *Theobroma cacao* [26–29]. Moreover, the *Gossypium* genus has experienced lineage-specific WGD, divergence and hybridization. As shown in Fig. 3a, we identified 33, 54, 25, 32, 63, 67, 107 and 107 *DIR* genes in *Vitis vinifera*, *Glycine max*, *Arabidopsis thaliana*, *Theobroma cacao*, *G. arboreum*, *G. raimondii*, *G. barbadense* and *G. hirsutum*, respectively. We assigned these *DIR* genes to distinct clades (Fig. 3b). Although they underwent different rounds of WGDs, these species were quite consistent in the number of DIR-a genes; *G. barbadense* and *G. hirsutum*, as tetraploid species, displayed DIR-a genes twice as many as the others. Similarly, apart from soybean, DIR-e was stable across species. Thus, DIR-a and DIR-e might have been set up before the divergence of Rosids, corresponding to the results inferred from the duplication events. In the above analysis, we found that DIR-f was evolutionarily inactive in *G. barbadense* and *G. hirsutum*. As expected, *G. arboreum*, *G. raimondii*, *Arabidopsis thaliana* and *Theobroma cacao*, belonging to Malvids, possessed less DIR-f genes. In particular, *Arabidopsis thaliana* lost the DIR-f clade during its evolution. DIR-b/d-I existed in all the eight species, DIR-b/d-II was absent in grape and soybean, and DIR-b/d-III was lineage-specific in cotton. Clearly, DIR-b/d-I, −II and -III really appeared in turn in evolution. It has been shown that DIR-b/d-III displayed two distinct clades and that the left clade arose later than the right. As shown in Fig. 3c, most genes in the left clade of DIR-b/d-III were in the collinear blocks, suggesting that DIR-b/d-III was established before the divergence of cotton species. The chromosome reciprocal translocation between At04 and At05 was also observed, which has been confirmed recently [30].

### Expression patterns and transcriptional regulation

**Fig. 4:**
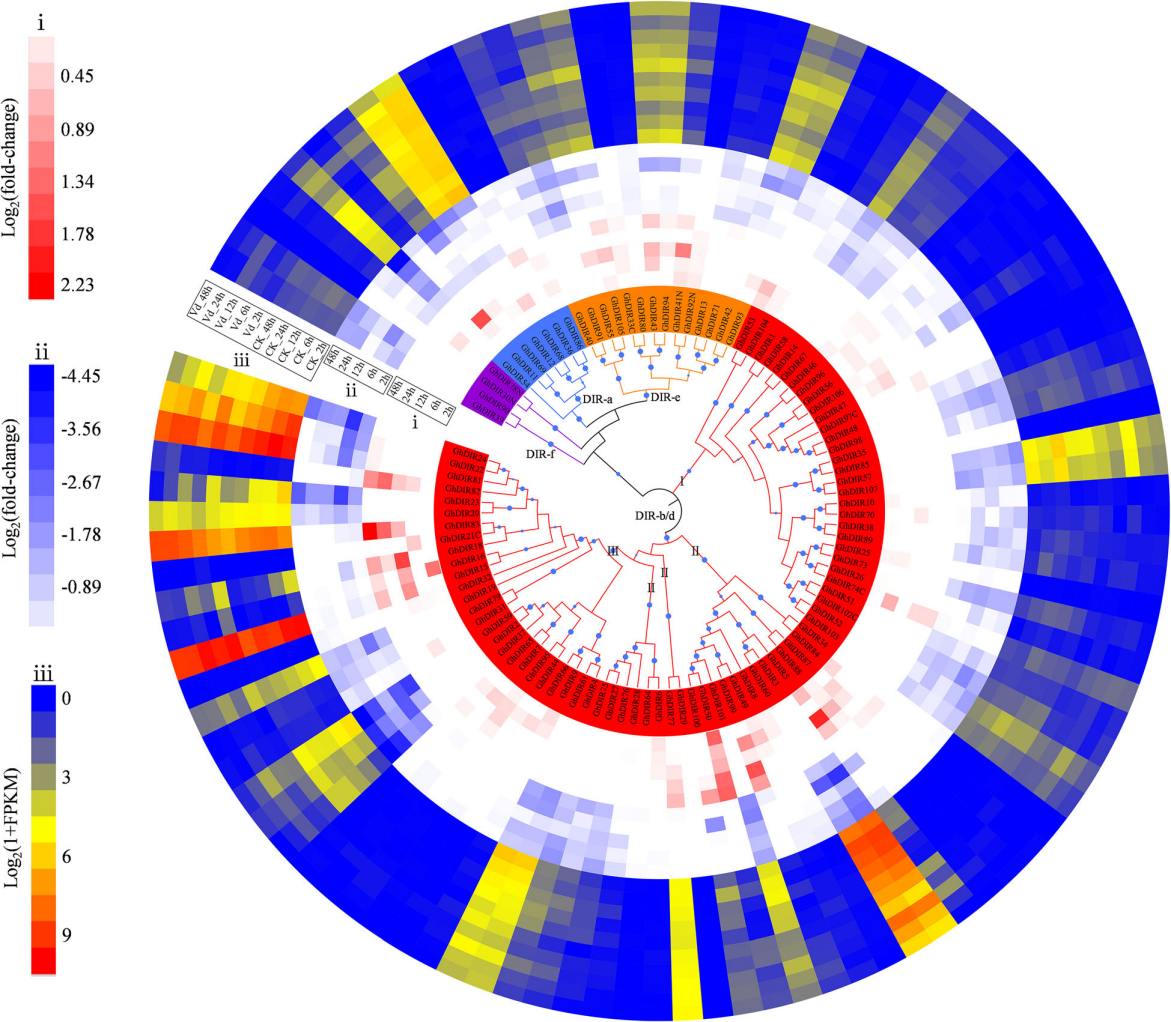
Circular heatmap depicting the expression patterns of *GhDIRs* in response to *V. dahliae* and water (CK). RNA-seq data were generated from the roots of four-week-old cotton seedlings. Panel i shows up-regulated genes after inoculation with *V. dahliae* (compared with the CK group), while panel ii shows down-regulated genes. Panel iii shows *V. dahliae*- and water-responsive expression levels. The original FPKM values were provided as Additional file 8: Table S11

Studies have shown that DIR proteins are implicated in lignan, lignin and gossypol biosynthesis, which are all part of plant defense responses against pathogens. To investigate the role of cotton *DIR* genes in disease resistance, the expression patterns of *GhDIRs* were analyzed in response to *V. dahliae* and water (the check group) using a Verticillium wilt-resistant cultivar. As a result, about one quarter of *GhDIRs* were highly expressed in the check group (Fig. 4). For the “gossypol clade” (the left half of DIR-b/d-III), almost half of the members showed a fairly high expression level, indicating its importance in plant pre-formed defense. Once the seedlings were inoculated with *V. dahliae*, most of the highly expressed *GhDIRs* were dramatically down-regulated. It seems that *V. dahliae* can weaken the functions of *DIR* genes by disturbing their expression to colonize cotton hosts. These down-regulated genes should be an important resource to understand plant-pathogen interaction. To verify the intriguing expression patterns, we also analyzed *V. dahliae*- and water-responsive expression of *GhDIRs* at 12 hpi in six other cultivars, and the log_2_(fold-change) values were presented in a heatmap (Additional file 4: Figure S4). After inoculation with *V. dahliae*, the cultivars S1 and S2 exhibited the largest number of down-regulated *DIR* genes, corresponding to their lowest Verticillium wilt resistance. In contrast with S1 and S2, the cultivars T3 and T4 showed more up-regulated genes in the DIR-a and DIR-e clades, and thus showed higher Verticillium wilt tolerance. Unlike T3, T4, S1 and S2, the cultivars T1 and T2 displayed quite a number of up-regulated *DIR* genes following inoculation with *V. dahliae*. Despite having different patterns in different cultivars, *DIR* genes might have contributed to cotton Verticillium wilt resistance.

**Fig. 5:**
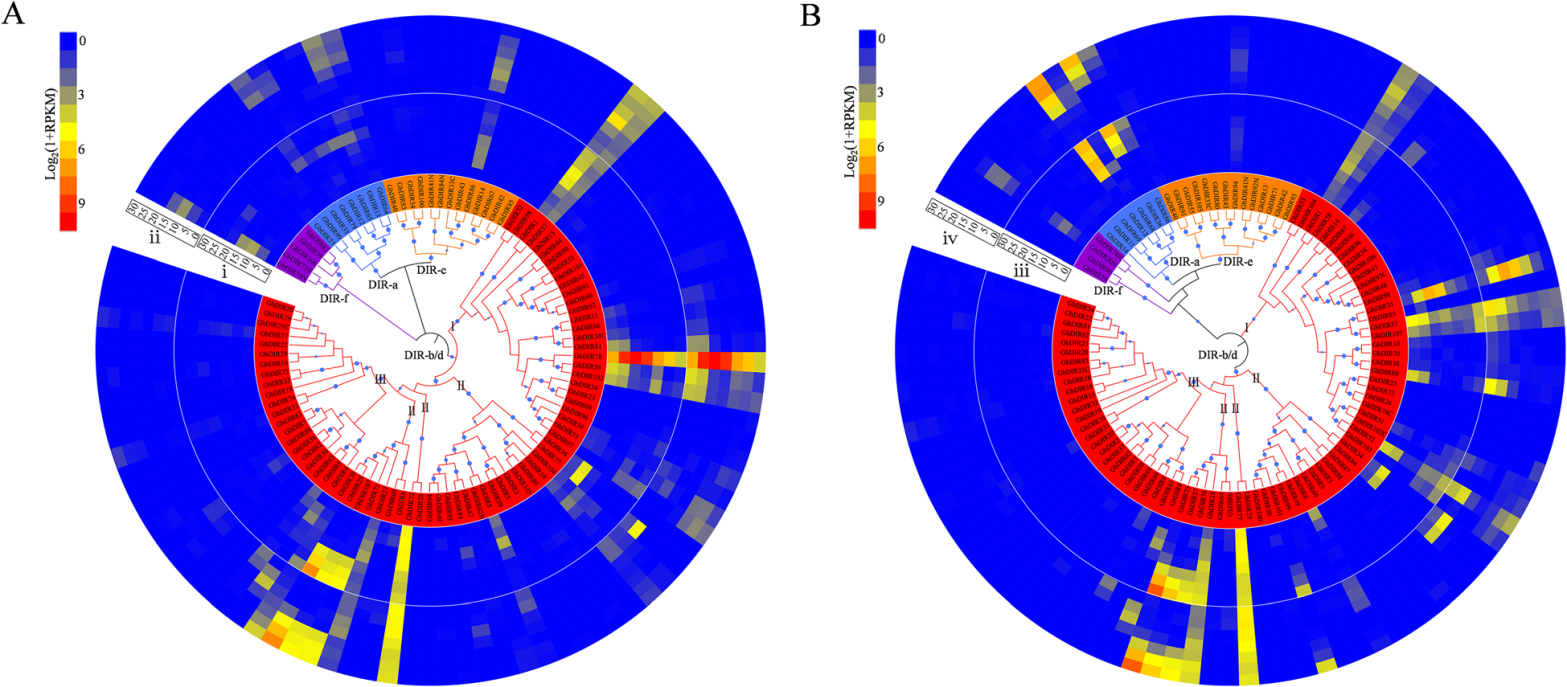
Expression patterns of *DIR* genes in cotton fibers. **a** Expression data from *G. barbadense* cultivars Pima90–53 (i) and Hai7124 (ii). **b** Expression data from *G. hirsutum* cultivars HY405 (iii) and ND13 (iv). 0–30 correspond to the number of days post-anthesis. The original RPKM values were provided as Additional file 8: Table S13 and S14

Considering that lignin/lignin-like phenolics can affect cotton fiber quality, we analyzed the expression patterns of *DIR* genes during cotton fiber development (Fig. 5). As shown, the expression profiles in *G. barbadense* (Pima90–53 and Hai7124) were similar to that in *G. hirsutum* (HY405 and ND13), exhibiting low expression levels in the DIR-e, DIR-f and DIR-b/d-III clades. Several genes belonging to DIR-b/d-II (*GbDIR25*, *GbDIR71* and *GbDIR107* in *G. barbadense*; *GhDIR27*, *GhDIR28*, *GhDIR75* and *GhDIR76* in *G. hirsutum*) were preferentially expressed in cotton fibers of 20, 25 and 30 DPA, indicating potential functions in secondary wall development. *GbDIR78* and *GhDIR35* which were part of DIR-b/d-I showed high transcript levels in the fibers of 5, 10 and 15 DPA, suggesting their importance during cotton fiber elongation. Furthermore, *GbDIR78* and *GhDIR35* were highly homologous but differentially expressed. Two DIR-a genes *GhDIR12* and *GhDIR36* were highly expressed during secondary wall thickening, whereas their orthologous genes *GbDIR13* and *GbDIR35* exhibited quite low expression levels. The differential expression might have contributed to the different fiber quality between these two species.

**Fig. 6:**
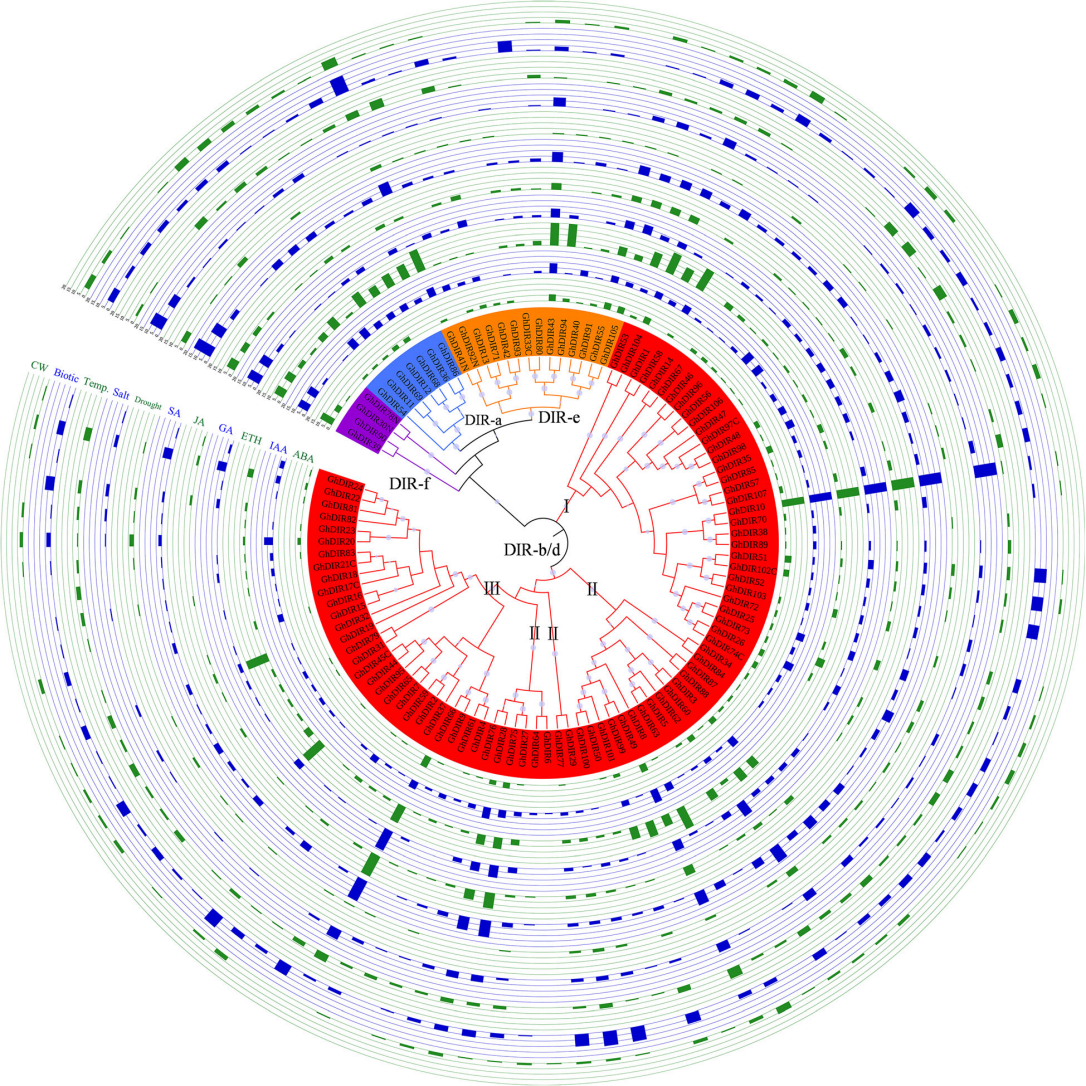
Identification of TFBS in the promoter regions of *GhDIRs*. A total of 266 JASPAR matrices were selected. FIMO was used to identify potential sites with a threshold of *p*-value < 1E-5. Histograms represent the number of TFBS in each category

Transcription factor binding sites (TFBS) provide cues for transcriptional regulation. A total of 266 JASPAR matrices were selected and fetched to identify potential TFBS in the promoter regions of *GbDIRs* and *GhDIRs* (Additional file 8: Table S8). Despite the biased JASPAR database and the strict threshold, TFBS were widely detected, including hormone-activated signaling pathway (ABA, IAA, ETH, GA, JA and SA), response to abiotic stresses (drought, salt and temperature), response to biotic stresses, and plant cell wall development (Fig. 6; Additional file 5: Figure S5; Additional file 8: Table S9 and S10). In the DIR-a, DIR-e and DIR-f clades, *GhDIR33*, *GhDIR36*, *GhDIR78*, *GhDIR80*, *GhDIR86* and *GhDIR92* had no TFBS related to ABA signal transduction and showed little or no expression in the roots of cotton seedlings (Fig. 4); the others tended to be highly expressed, indicating the probable regulatory roles of ABA signaling in root-specific gene expression. Four DIR-b/d-II genes *GhDIR27*, *GhDIR28*, *GhDIR75* and *GhDIR76* were highly expressed in cotton fibers. Despite having extremely close phylogenetic relationships with these four genes, *GhDIR6* and *GhDIR64* exhibited quite low expression levels (Fig. 5b). One cause may be the lack of TFBS in their promoter regions (Fig. 6). Similarly, *GbDIR6* and *GbDIR60* differed from *GbDIR25*, *GbDIR71* and *GbDIR107* in TFBS occurrences and in transcript levels (Fig. 5a; Additional file 5: Figure S5). As another example, *GhDIR36* carried more IAA-responsive TFBS than *GbDIR35*, corresponding to their differential expression in cotton fibers (Fig. 5; Additional file 6: Figure S6a). Considering that *GbDIR13* and *GhDIR12* differed in transcript levels in cotton fibers but owned similar TFBS (Fig. 5; Additional file 6: Figure S6b), their *trans*-acting TFs were analyzed. As shown, some TFs associated with IAA signaling, ETH signaling or plant cell wall development exhibited higher expression levels in *G. hirsutum* than in *G. barbadense*.

### Functional characterization of *GbDIR78* in *Arabidopsis*

**Fig. 7:**
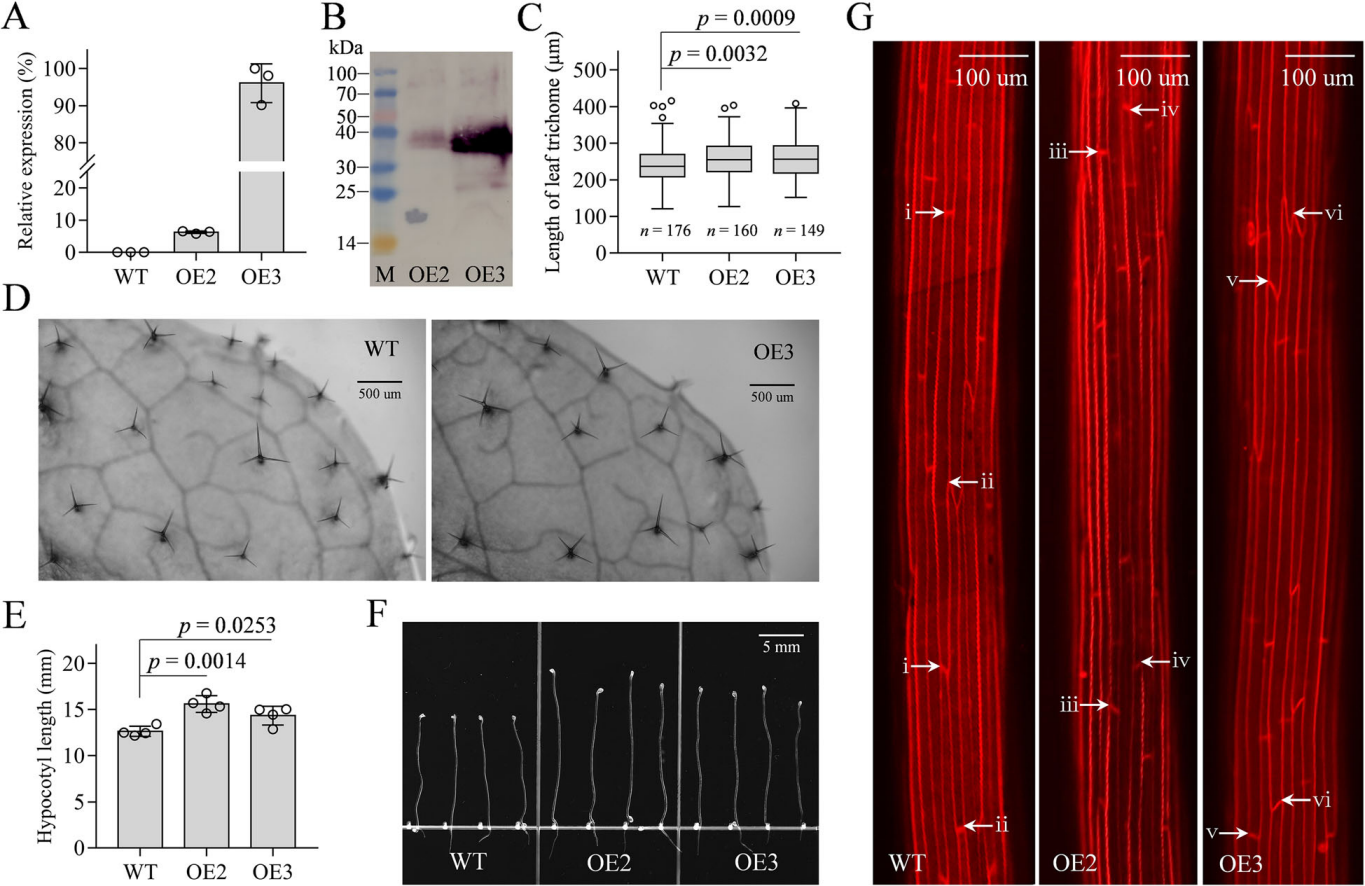
Functional characterization of *GbDIR78* in *Arabidopsis*. **a** Expression of *GbDIR78* in the transgenic *Arabidopsis*, tested with qRT-PCR. **b** Western blot of GbDIR78 in the transgenic *Arabidopsis*. **c-d** *Arabidopsis* with overexpression of *GbDIR78*, compared with wild type, showed longer leaf trichomes. In the box plots, the median, upper and lower quartiles, and the range of normal values are denoted by the center line, box limits, and whiskers, respectively. *n* indicates the number of measured trichomes. The significance of difference was analyzed with two tailed *t* test. **e-f** *Arabidopsis* with overexpression of *GbDIR78*, compared with wild type, showed significantly longer dark-grown hypocotyls. **g** Microscopic inspection of hypocotyl epidermal cells. i-vi indicate the ends of epidermal cells. The original, unprocessed images were provided as Additional file 9

RNA-seq data showed *GbDIR78* was preferentially expressed during cotton fiber elongation and that *GbDIR78* and *GhDIR35* differed in transcript levels, implying the involvement of *GbDIR78* in cell elongation. To further identify its functions, the ORF of *GbDIR78* driven by a 35S promoter was transformed into *A. thaliana* plants. Two transgenic T_3_ lines OE2 and OE3 were generated, and the stable expression of *GbDIR78* was confirmed by Real-time PCR and Western blot (Fig. 7a and b). *Arabidopsis* leaf trichomes can serve as a useful experimental system to dissect cotton fiber development because they partly share regulatory mechanisms [31–34]. Here, trichomes from the fifth rosette leaves of OE2, OE3 and WT plants were measured, and then we discovered that the transgenic plants owned significantly longer trichomes (Fig. 7c and d). Moreover, dark-grown hypocotyls were utilized to determine the role of *GbDIR78* in cell elongation because their growth resulted from cell elongation rather than division [35, 36]. As a result, the seedlings of OE2 and OE3, compared with WT seedlings, showed significantly longer hypocotyls (Fig. 7e and f). As expected, the longer epidermal cells were observed in the hypocotyls of transgenic plants in a microscopic inspection (Fig. 7g). These results indicate that *GbDIR78* can promote cell elongation and might have contributed to cotton fiber development.

## Discussion

### Type b/d group expanded considerably and evolved rapidly in cotton

Gene duplication events (tandem, segmental/whole-genome or by transposition) have provided raw evolution materials and meanwhile built up various types of gene families. Among them, tandem and segmental duplications (from unequal crossing-over and infrequent polyploidy, respectively) are fully thought out in the evolution of plant gene families [37, 38]. In the present study, the expansion of DIR-b/d was mainly due to tandem duplications (Fig. 2 and Table 1). As a result, a number of gene clusters were generated, and the chromosomes At01, At04, At11, Dt01, Dt04 and Dt11 contained only DIR-b/d genes (Additional file 2: Figure S2). Interestingly, the heterogeneous gene clusters including DIR-b/d-II and DIR-b/d-III members were observed on chromosomes At01 and Dt01, which provided clues about the initial generation of DIR-b/d-III. DIR-b/d genes accounted for 75.7% in *G. barbadense* and 76.6% in *G. hirsutum*, while 34.3, 20.4, 50.0 and 56.8% in spruce, rice, pepper and flax, respectively [15, 17, 19, 21]. Also, as shown in Fig. 3b, the proportion of DIR-b/d genes in cotton was much larger than that in *Vitis vinifera*, *Glycine max*, *Arabidopsis thaliana* and *Theobroma cacao*. All these results showed a *Gossypium*-specific expansion of DIR-b/d. Given the rare segmental duplication events in plant genomes, tandem duplication has been proposed as a proper mechanism to cope with rapidly changing environments [39, 40]. Therefore, the rapid expansion of DIR-b/d might be a plant adaptive evolution against biotic and abiotic stresses. For example, a cluster of high-density genes located on chromosome At04 was involved in gossypol biosynthesis (Additional file 2: Figure S2) and might have contributed to plant defense responses against pathogens and pests. Moreover, tandem duplication acts different contribution across species; tandemly duplicated *DIR* genes are abundant in *Medicago truncatula* and *Oryza sativa* but scarce in *Capsicum annuum* [15, 19, 20]. To explore why tandem duplications promoted the rapid expansion of DIR-b/d, we compared the strength of selection acting on tandemly duplicated genes (Additional file 3: Figure S3c and S3d). Compared with the genes in DIR-a and DIR-e, tandemly duplicated genes in DIR-b/d, especially DIR-b/d-III, showed larger Ka/Ks values, which meant weaker purifying selection and greater evolutionary rates. In other words, the relaxed selective constraints might have accelerated neofunctionalization corresponding to the occurrence of “gossypol clade”. Moreover, this may be one reason for the rapid expansion of cotton DIR-b/d subfamily. Considering that the calculated Ka/Ks values were averaged over sites and time, positive selection might have worked at individual sites or in a short period, which benefitted the retention of gene duplicates. In brief, perhaps to cope with environmental challenges, the DIR-b/d clade expanded considerably and evolved rapidly in allotetraploid cotton. The tandemly arrayed *DIR* genes should also be candidate resistance resources in breeding programs.

### DIRs may substantially affect cotton fiber development

Lignin, deposited mostly in the secondary cell walls of vascular plants, contributes to water transport, mechanical support and plant stress responses. Besides, the deposition of lignin in cell walls can repress cell growth due to the decreased extensibility. In cotton fibers, lignin has been neglected for the low concentrations. However, in recent years, studies suggest that lignin-like phenolics may substantially affect cotton fiber quality [41–43]. Given the involvement of *DIR* genes in lignin biosynthesis, we analyzed their expression patterns during cotton fiber development. A DIR-b/d-II “SCW clade” was observed in *G. hirsutum*, consisting of *GhDIR27*, *GhDIR28*, *GhDIR75* and *GhDIR76* (Fig. 5b). Being highly expressed during secondary wall thickening, they might affect lignin deposition in cotton fibers. After analyzing QTLs reported for fiber quality traits, *GhDIR27* and *GhDIR28* fell in qFS-c4–1, a stable QTL across multiple environments controlling fiber strength [44]. Also, two other QTLs for fiber strength (qFS-C4–3 and qFS04.1) and one QTL controlling fiber micronaire (qFM-Chr4–3) were detected at almost the same region [45–47]. Interestingly, *GhDIR27* and *GhDIR28* also fell in qFL04.1, a QTL conferring fiber length [48]. Thus, it is reasonable to speculate that this clade might regulate lignin biosynthesis and affect fiber development. Similarly, “SCW clade” was also observed in *G. barbadense* (Fig. 5a). However, *GbDIR27* and *GbDIR70* (orthologous to *GhDIR28* and *GhDIR75*, respectively) showed quite low expression levels, which might explain in part different fiber properties between the two cotton species. DIR-a genes are widely considered to mediate lignan biosynthesis (Additional file 8: Table S6). In particular, a soybean DIR-a protein GmPdh1 was inferred to affect the pattern of lignin deposition by considering its expression patterns and its promotion to pod dehiscence [16]. It reminds us of that the loss-of-function mutation of *AtPrR1* (pinoresinol reductase) results in alterations in lignin levels, lignin structure and tissue-specific lignin distribution [49]; there may be an association between lignan biosynthesis and lignin deposition. In the present study, two DIR-a genes *GhDIR12* and *GhDIR36* broadly similar to *GmPdh1* were highly expressed during secondary cell wall thickening (Fig. 5b). However, their orthologous genes (*GbDIR13* and *GbDIR35*, respectively) exhibited little expression in cotton fibers (Fig. 5a). It is interesting to investigate whether *GhDIR12* and *GhDIR36* can negatively regulate fiber quality by enhancing lignin biosynthesis. Unlike the above-mentioned genes, *GhDIR35* was preferentially expressed during fiber elongation. It belonged to the DIR-b/d-I clade and fell in qFL08.1, a stable QTL for fiber length [50]. *GbDIR78*, highly homologous to *GhDIR35*, showed a higher transcript level. Moreover, the overexpression of *GbDIR78* in *Arabidopsis* plants can promote cell elongation (Fig. 7). Therefore, *GbDIR78* and *GhDIR35* may play an important role in cotton fiber elongation, and their differential expression might have contributed to the different fiber properties.

### *GbDIR78* promotes cell elongation possibly by regulating phenylpropanoid metabolism

*GbDIR78* can promote cell elongation in *Arabidopsis* plants, but the mechanisms need to be discussed. Being preferentially expressed during cotton fiber elongation, *GbDIR78* is not likely able to participate in lignin biosynthesis. A soybean gene *GmDIR22* can effectively direct lignan biosynthesis in vitro and in vivo [8]. *GbDIR78* was quite close to *GmDIR22* in phylogenetic tree (Fig. 2c), implying that *GbDIR78* may also be involved in lignan biosynthesis. Moreover, when transiently expressed in onion epidermal cells, GbDIR78 entered the secretory pathway and was mainly retained in the plasma membrane (Additional file 7: Figure S7), which is partly similar to the subcellular localization of GmDIR22. However, DIR proteins are targeted to the cell wall when involved in lignin biosynthesis [9, 10]. Thus, we reasonably speculate that *GbDIR78* participates in lignan biosynthesis. Slightly confusingly, the reported cotton *GhDIR1* gene shares close evolutional relationships with *GbDIR78*, but the overexpression of *GhDIR1* in cotton can enhance lignification [11]. This could be explained in terms of the association between lignan and lignin biosynthesis [49].

Apoplast ROS (reactive oxygen species) signaling is crucial for cell elongation [51], but at high concentrations ROS become toxic, causing cell wall stiffening [52]. The cell walls of *Arabidopsis* plants overexpressing *GbDIR78* may be at moderate ROS concentrations, because some (neo)lignans can act against oxidative damage [53]. Given that lignans compete with flavonoids for phenylalanine precursors [54, 55], the metabolic flux towards lignans can result in a reduction of flavonoid biosynthesis. Flavonoids (especially flavonols) have been shown to inhibit polar auxin transport [56]. Thus, in *GbDIR78*-overexpressed *Arabidopsis* plants, auxin transport (in an apical-basal axis) may be elevated compared with WT plants. To sum up, the longer trichomes and hypocotyls in the transgenic *Arabidopsis* plants might be due to moderate ROS levels and higher auxin accumulation. In cotton fibers, appropriate ROS levels are important for cell elongation [57, 58]. Also, some flavonoids play a negative impact on cotton fiber development [59]. Thus, the metabolic flux from flavonoids into lignans should be a novel alternative way to improve cotton fiber quality.

## Conclusions

In summary, we performed a genome-wide analysis of *DIR* gene family in *G. barbadense* and *G. hirsutum*. Our study clearly demonstrates how segmental and tandem duplications contribute to the expansion of cotton *DIR* gene family and highlights a *Gossypium*-specific clade involved in atropselective synthesis of gossypol. We also suggest that *DIR* genes can not only confer Verticillium wilt resistance but also affect cotton fiber development. In addition, the fact that *GbDIR78* can promote cell elongation in *Arabidopsis* plants paves an alternative way to improve cotton fiber properties. Our results provide useful insights into the evolutionary history, expression patterns, transcriptional regulation, and functional analysis of *Gossypium DIR* genes.

## Methods

### Plant materials and growth conditions

*G. barbadense* cv. Pima90–53 [60] and Hai7124 [61], with superior fiber quality, and *G. hirsutum* cv. HY405 and ND13, with moderate fiber quality, were grown at an experimental field in Baoding (38°45′N, 115°29′E) during the growing season (late April to late October). Field management followed routine farming methods. *G. hirsutum* cultivars with different resistance to *V. dahliae* infection used in this study are as follows: a resistant cultivar ND601 [62], four tolerant cultivars (AusSiV2, Xinmian33B, Nongdamian7 and Nongdamian8), two susceptible cultivars (Handan333 and Xiangmian18). The Verticillium wilt resistance was assessed on the basis of observations at a disease nursery over several years. In *V. dahliae*-responsive expression experiments, the seedlings of these cultivars were grown in 50% Hoagland’s solution under environmental conditions of 28 °C/25 °C (day/night), 16-h photoperiod, and 80% relative humidity. The solution was changed every four days in order to ensure the healthy growth of seedlings. Four-week-old cotton seedlings were infected with *V. dahliae* strain Linxi2–1 as described by Wang et al. [63]. Most of the above-mentioned cultivars were collected and preserved, with the appropriate permissions, by the National Medium-term Gene Bank of Cotton in China, including Pima90–53 (M210080, introduced from USA), Hai7124 (M210054; Jiangsu, China), AusSiV2 (M131662, introduced from Australia), Xinmian33B (M112566, introduced from USA), Nongdamian7 (M110598; Hebei, China), Nongdamian8 (M110599; Hebei, China), Handan333 (M112751; Hebei, China) and Xiangmian18 (M114752; Hunan, China). The other three cultivars HY405, ND13 and ND601 were collected, from Hebei Province in China, by Hebei Agricultural University, and their accession numbers were G100937, G100728 and G100729, respectively. All necessary permissions for planting and investigating these cultivars were obtained from Hebei Agricultural University and the National Medium-term Gene Bank of Cotton in China, and the collection and research of these cultivars have complied with the Convention on the Trade in Endangered Species of Wild Fauna and Flora. *A. thaliana* Columbia wild-type plants (Col-0) and transgenic plants were cultivated in pots containing sterile vermiculite in a greenhouse (22 °C, 16-h photoperiod, and 70% relative humidity). Hoagland’s nutrient solution was added weekly.

### Sequence sources

The genomic data of *G. raimondii* (JGI_221_v2.1) [64], *G. arboretum* (CRI_v2) [65], *G. hirsutum* (ZJU_TM-1_V2.1) and *G. barbadense* (ZJU_Hai7124_V1.1) [61] were downloaded from CottonFGD [66]. The *V. vinifera* (12X) [27] and *T. cacao* (Criollo_cocoa_genome_V2) [29] genomes were downloaded from Ensembl Plants [67], and the *G. max* (Wm82.a2.v1) [28] genome was downloaded from Phytozome [68]. The *DIR* genes of above-mentioned species were identified in this study. The DIR sequences of *A. thaliana* [23], *L. usitatissimum* [21], *O. sativa* [19] and *Picea* [17] were retrieved from TAIR (http://www.arabidopsis.org/), Phytozome (https://phytozome.jgi.doe.gov/pz/portal.html), Rice Genome Annotation Project (http://www.rice.plantbiology.msu.edu/), and NCBI (http://www.ncbi.nlm.nih.gov/), respectively.

### Identification and characterization of DIR proteins

The sequence alignments of *DIR* gene family (PF03018) were downloaded from Pfam [69]. The candidate DIR proteins were identified using HMMER 3.0 [70] and confirmed with the Batch CD-Search service [71]. N-glycosylation sites were identified using NetNglyc 1.0 (http://www.cbs.dtu.dk/services/NetNGlyc/). Signal peptide prediction was performed with SignalP 5.0 [72]. YLoc served for predicting subcellular localization [73]. The protein length, molecular weight and isoelectric point were investigated by a native Perl script.

### Gene structure, chromosomal distribution, conserved motifs and phylogenetic analysis

The exon-intron information and gene location information were fetched from gene annotation files and subsequently visualized using TBtools [74]. MEME was employed to search conserved motifs, with a limit of 15 motifs and other default parameters [75]. The phylogenetic trees were constructed by MEGA 7.0 using the neighbor-joining (NJ) method with 1000 bootstrap replications [76] and then displayed with the online iTOL tool [77].

### Gene duplication and the calculation of Ka, Ks and Ka/Ks values

Segmental and tandem duplications were detected by MCScanX with default parameters [78]. The duplication events were fetched and then displayed with TBtools [74]. Homologous genes between At- and Dt-subgenome were determined using the bidirectional best hit method in BLAST. We used ParaAT [79] to construct protein-coding DNA alignments. Then the paired sequences were used to calculate Ka, Ks and Ka/Ks values using KaKs_Calculator with the LPB method [80].

### RNA-seq data

A Verticillium wilt-resistant *G. hirsutum* cultivar ND601 and a highly aggressive defoliating *V. dahliae* strain Linxi2–1 were used in *V. dahliae*-responsive expression analysis. The roots of four-week-old seedlings infected by *V. dahliae* were collected independently at 2, 6, 12, 24 and 48 h post-inoculation (hpi), while the roots of seedlings inoculated with distilled water, as control, were also collected at the corresponding time points. For each time point, two biological replicates were generated. Frozen roots were ground mechanically to a fine powder in liquid nitrogen. Then total RNA was isolated using an RNAprep pure Plant Kit (TIANGEN, Beijing, China), following the manufacturer’s instructions. For RNA-seq, strand-specific cDNA libraries were prepared at the Novogene Bioinformatics Institute, Beijing, China. An Illumina Hiseq 4000 platform was then used for sequencing, and 150 bp paired-end reads were generated. Then gene expression levels were calculated using FPKM (Fragments Per Kilobase of exon model per Million mapped reads). Finally, log_2_(1 + FPKM) values after averaging two replicates were displayed. Similarly, four Verticillium wilt-tolerant *G. hirsutum* cultivars AusSiV2, Xinmian33B, Nongdamian7 and Nongdamian8 (termed as T1, T2, T3 and T4, respectively, during the current study) and two susceptible cultivars Handan333 and Xiangmian18 (termed as S1 and S2, respectively) were used for expression experiments. RNA-seq samples were generated at 12 hpi in the same way described above. To highlight expression changes, we showed fold-change values with log_2_ transformation. The fold change is the ratio of 1 + FPKM (treatment) to 1 + FPKM (control). In addition, *G. barbadense* cultivars Pima90–53 and Hai7124, and *G. hirsutum* cultivars HY405 and ND13 were employed in fiber development-related expression analysis. For each cultivar, cotton bolls were harvested independently at 0, 5, 10, 15, 20, 25 and 30 days post-anthesis (DPA). For each time point, samples from multiple cotton bolls were collected and pooled to minimize variations. Finally, 28 libraries (generated from ovules of 0 DPA, and fibers of 5, 10, 15, 20, 25 and 30 DPA) were used to produce 125 bp paired-end reads on a HiSeq 2500 platform. RPKM (Reads Per Kilobase of exon model per Million mapped reads) was applied to estimate expression levels, and we finally showed log_2_(1 + RPKM) values.

### Promoter analysis

The promoter sequences (2000-bp upstream of ATG) of *DIR* genes were extracted from genomes of *G. barbadense* and *G. hirsutum*. According to Gene Ontology annotations, a total of 266 JASPAR matrices (transcription factor binding profiles) were selected and then fetched, including hormone-activated signaling pathway (ABA, IAA, ETH, GA, JA and SA), response to abiotic stresses (drought, salt and temperature), response to biotic stresses, and plant cell wall development [81]. For each JASPAR matrix, FIMO was used to scan promoter sequences for matches with a strict threshold of *p*-value <1E-5 [82]. The potential transcription factor binding sites (TFBS) were counted according to their classification information. Finally, we showed the results using iTOL [77].

### Functional analysis of *GbDIR78*

The coding sequence of *GbDIR78* (from Pima90–53) was amplified and inserted into the Gateway pDONR207 vector to form an entry clone. Then the coding sequence was recombined into the Gateway pGWB414 vector to generate an overexpression (OE) construct under the control of CaMV 35S promoter. The OE construct was transformed into *A. thaliana* (Col-0) through *Agrobacterium*-mediated plant transformation. Transgenic plants were identified using 50 μg/ml kanamycin screening (1/2 MS medium) and PCR detection. Two transgenic T_3_ lines OE2 and OE3 were generated, and the stable expression of *GbDIR78* was confirmed by Real-time PCR and Western blot (HA-Tag). Decolorized by ethanol, the fifth rosette leaves of four-week-old wild type (WT) and OE plants were photographed with an Olympus BX51 microscope (Tokyo, Japan), and the longest branch was measured using the ImageJ software for each of about 150 legible trichomes in each line [83]. To observe dark-grown hypocotyls, the seeds of *A. thaliana* were sterilized and then grown in vertical plates (1/2 MS medium, 0.9% agar and pH 5.8) under the conditions of 22 °C and continuous darkness. Five-day-old WT and OE seedlings were harvested and then photographed with a professional Epson V800 scanner (Nagano, Japan). Their hypocotyls were subsequently measured using the ImageJ software [83]. To observe the epidermal cells of the hypocotyls, the seedlings used above were stained with propidium iodide (PI) and then photographed using an Olympus FV10i laser scanning microscope (Tokyo, Japan). Two-tailed *t* test (*p*-value) was conducted using the GraphPad Prism software (San Diego, CA, USA). To examine the subcellular localization of GbDIR78, its coding sequence was recombined into the Gateway pEarleyGate103 vector, which can express target protein with a C-terminal GFP fusion. A plasmid expressing GFP alone served as a control. GbDIR78-GFP fusion protein and GFP were transiently expressed in onion epidermal cells by a Bio-Rad PDS-1000/He system (Hercules, CA, USA). Then the transformed cells were monitored with an Olympus BX51 microscope (Tokyo, Japan) after incubating on MS agar medium for 24 h (22 °C, continuous darkness).

## Supplementary Information

**Additional file 1: Figure S1**. Phylogenetic relationships, motif analysis and gene structure of *GbDIRs* (**a**) and *GhDIRs* (**b**). Fifteen distinct motifs were identified with MEME software. Exons and introns are represented by green boxes and black lines, respectively. The conserved domain regions are colored in yellow

**Additional file 2: Figure S2**. Chromosomal distribution of *GbDIRs* (**a**) and *GhDIRs* (**b**). Tandemly duplicated genes are colored in red and linked by red lines

**Additional file 3: Figure S3**. Ka/Ks values for segmental duplications among *GbDIRs* (**a**) and *GhDIRs* (**b**), and for tandem duplications among *GbDIRs* (**c**) and *GhDIRs* (**d**). Tandemly duplicated DIR-b/d-III genes are colored in red, while DIR-a and DIR-e genes are colored in blue

**Additional file 4: Figure S4**. Expression patterns of *GhDIRs* from Verticillium wilt tolerant and susceptible cultivars (**a-f**). Red boxes indicate up-regulated genes after inoculation with *V. dahliae* (compared with the CK group), while blue boxes indicate down-regulated genes. The figures in boxes represent log_2_(fold-change) values corresponding to color gradients. (**g**) The disease index at 20 dpi. T and S represent tolerant and susceptible *G. hirsutum* cultivars, respectively. The original FPKM values were provided as Additional file 8: Table S12

**Additional file 5: Figure S5**. Identification of TFBS in the promoter regions of *GbDIRs*

**Additional file 6: Figure S6**. The potential mechanisms causing the differential expression. (**a**) *GhDIR36* carried more IAA-responsive TFBS than *GbDIR35*. (**b**) Although *GbDIR13* and *GhDIR12* carried similar TFBS, the *trans*-acting TFs exhibited higher expression levels in *G. hirsutum* than in *G. barbadense*

**Additional file 7: Figure S7**. Subcellular localization of GFP alone or GbDIR78-GFP fusion protein in onion epidermal cells

**Additional file 8: Table S1**. Characteristics of 107 GbDIR proteins. **Table S2**. Characteristics of 107 GhDIR proteins. **Table S3**. List of *DIR* genes used in the phylogenetic tree. **Table S4**. Ka, Ks and Ka/Ks values calculated for homologous *DIR* gene pairs in *Gossypium barbadense*. **Table S5**. Ka, Ks and Ka/Ks values calculated for homologous *DIR* gene pairs in *Gossypium hirsutum*. **Table S6**. List of DIRs with biochemical and/or physiological functions in literatures. **Table S7**. List of *DIR* genes identified in *Gossypium arboreum*, *Gossypium raimondii*, *Glycine max*, *Theobroma cacao* and *Vitis vinifera*. **Table S8**. JASPAR matrices used to identify potential TFBS. **Table S9**. List of motif occurrences in *Gossypium barbadense*. **Table S10**. List of motif occurrences in *Gossypium hirsutum*. **Table S11**. FPKM values of *DIR* genes in response to *Verticillium dahliae* and water in *Gossypium hirsutum* cultivar ND601. **Table S12**. FPKM values of *DIR* genes in response to *Verticillium dahliae* and water in six *Gossypium hirsutum* cultivars. **Table S13**. RPKM values of *DIR* genes during cotton fiber development in *Gossypium barbadense*. **Table S14**. RPKM values of *DIR* genes during cotton fiber development in *Gossypium hirsutum*

**Additional file 9.** The original, unprocessed images

## Data Availability

The data that support the findings of this study are included in this published article and its additional files. The RNA-seq data are available in the Genome Sequence Archive (https://bigd.big.ac.cn/gsa; accession number: CRA003811, CRA003789 and CRA002927) or from the corresponding author on reasonable request.

## Abbreviations

MYA: Million years ago
Ks: Synonymous substitutions per synonymous site
Ka: Nonsynonymous substitutions per nonsynonymous site
WGD: Whole-genome duplication
TFBS: Transcription factor binding sites
ABA: Abscisic acid
IAA: Indole-3-acetic acid
ETH: Ethylene
GA: Gibberellic acid
JA: Jasmonic acid
SA: Salicylic acid
WT: Wild type
OE: Overexpression
ROS: Reactive oxygen species
hpi: hours post-inoculation
DPA: Days post-anthesis
QTL: Quantitative trait locus
