# Supplementary figures and images for: Evolution, expression and functional analysis of cultivated allotetraploid cotton DIR genes
Source: BMC Plant Biol. 2021 Feb 10;21:89. doi: 10.1186/s12870-021-02859-0 (PMC7876823; doi:10.1186/s12870-021-02859-0)

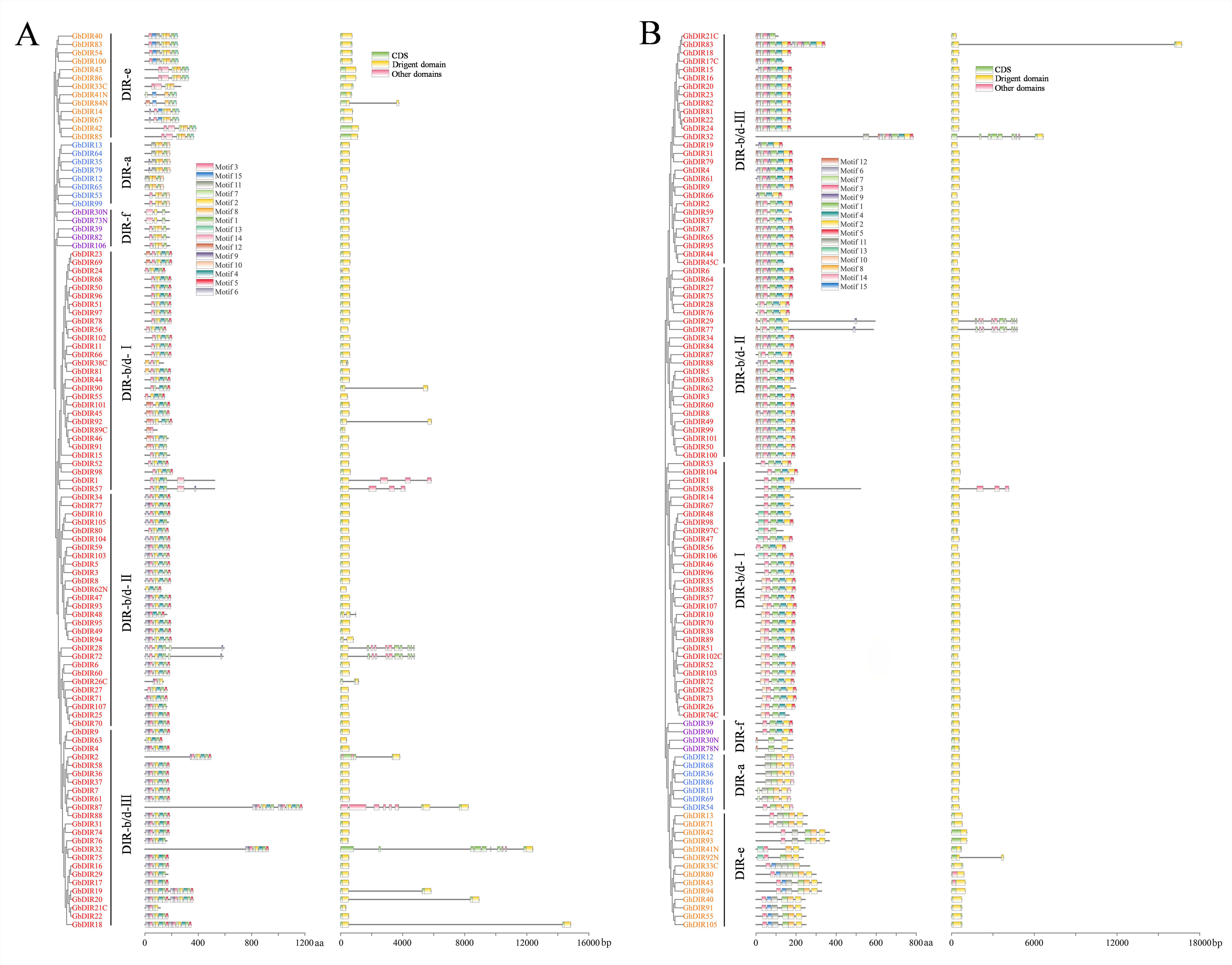

Supplement: Supplementary file 1 — Additional file 1: Figure S1. Phylogenetic relationships, motif analysis and gene structure of GbDIRs (a) and GhDIRs (b). Fifteen distinct motifs were identified with MEME software. Exons and introns are represented by green boxes and black lines, respectively. The conserved domain regions are colored in yellow [file 12870_2021_2859_MOESM1_ESM.tif]

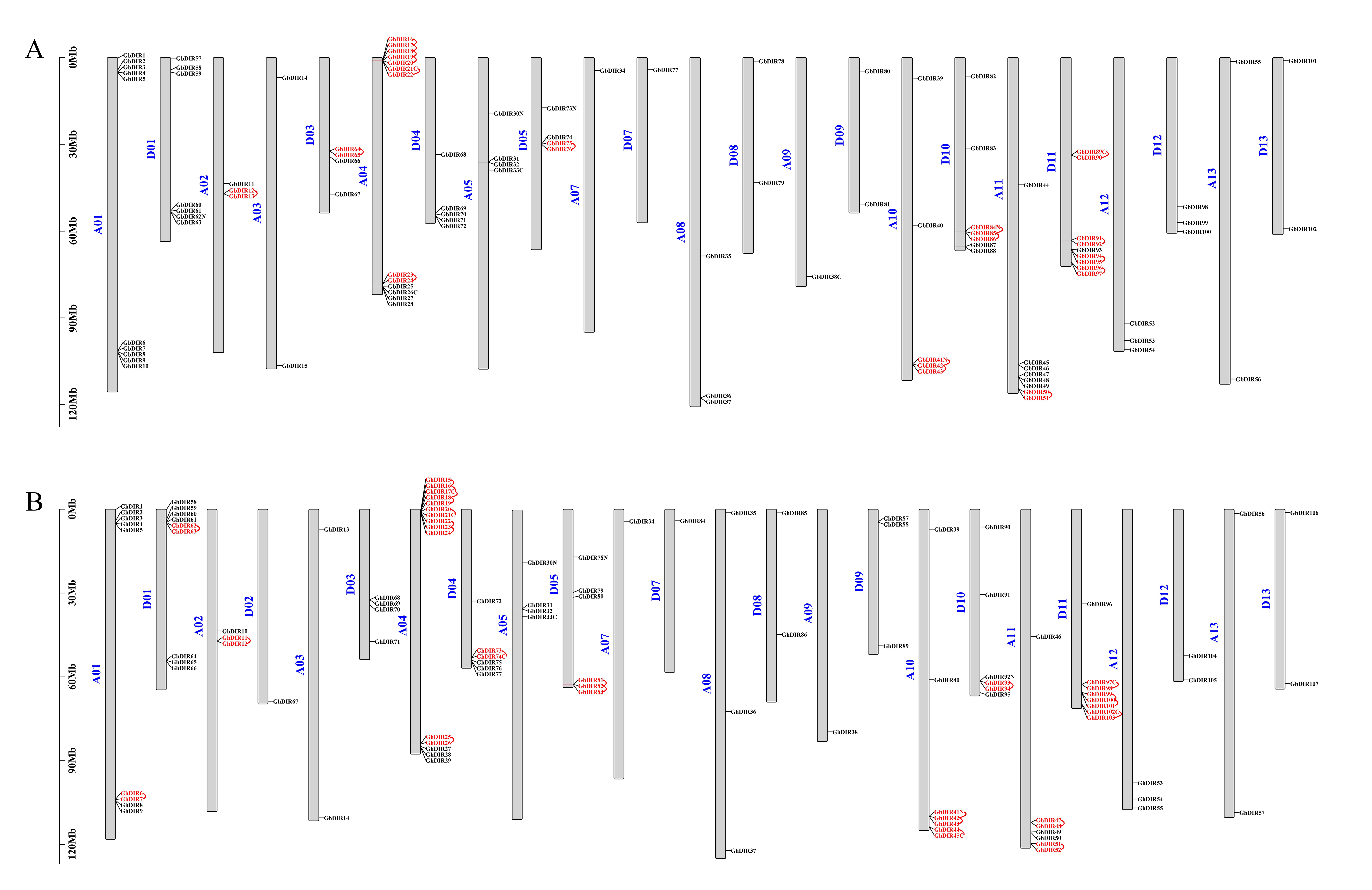

Supplement: Supplementary file 2 — Additional file 2: Figure S2. Chromosomal distribution of GbDIRs (a) and GhDIRs (b). Tandemly duplicated genes are colored in red and linked by red lines [file 12870_2021_2859_MOESM2_ESM.tif]

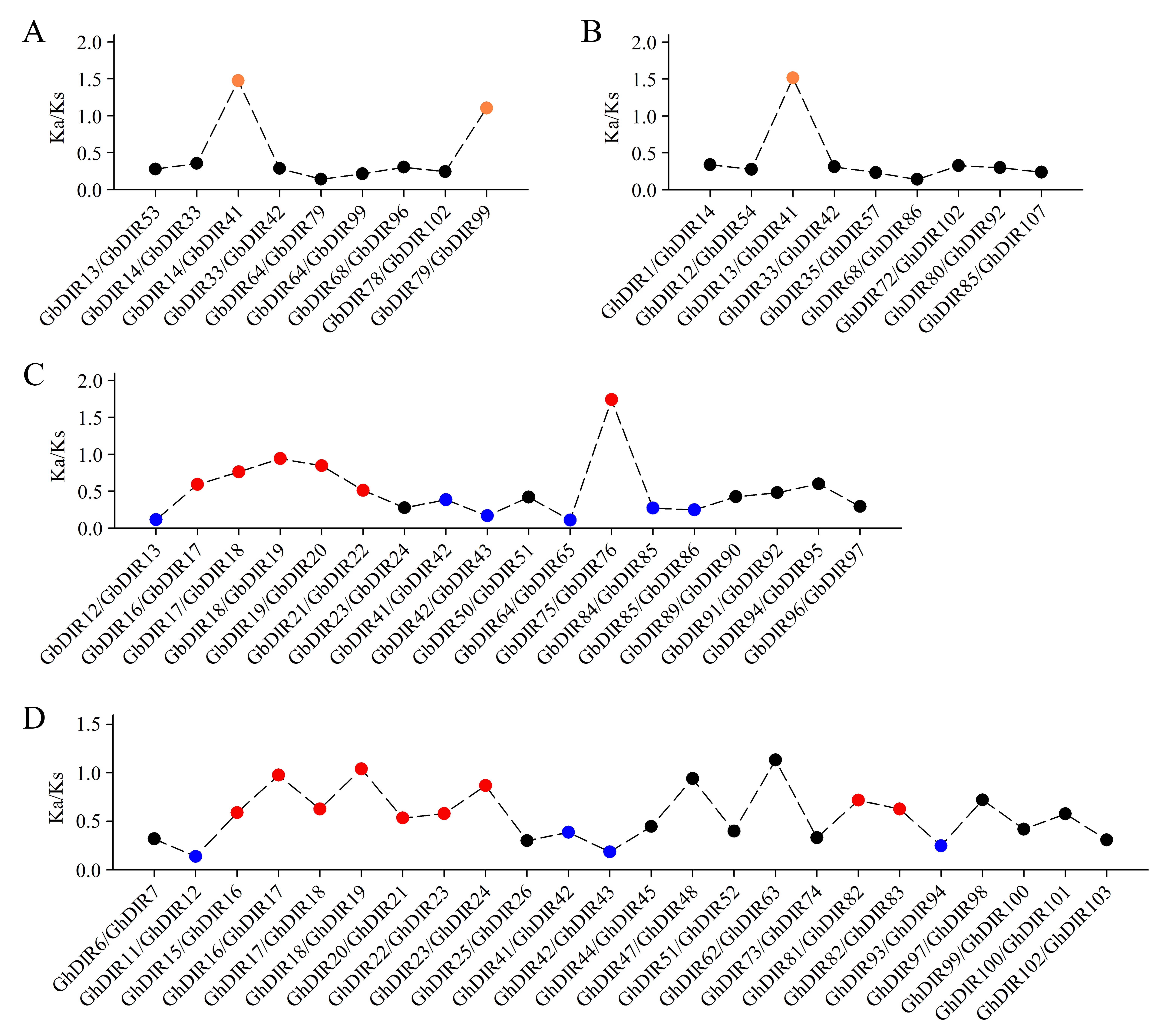

Supplement: Supplementary file 3 — Additional file 3: Figure S3. Ka/Ks values for segmental duplications among GbDIRs (a) and GhDIRs (b), and for tandem duplications among GbDIRs (c) and GhDIRs (d). Tandemly duplicated DIR-b/d-III genes are colored in red, while DIR-a and DIR-e genes are colored in blue [file 12870_2021_2859_MOESM3_ESM.tif]

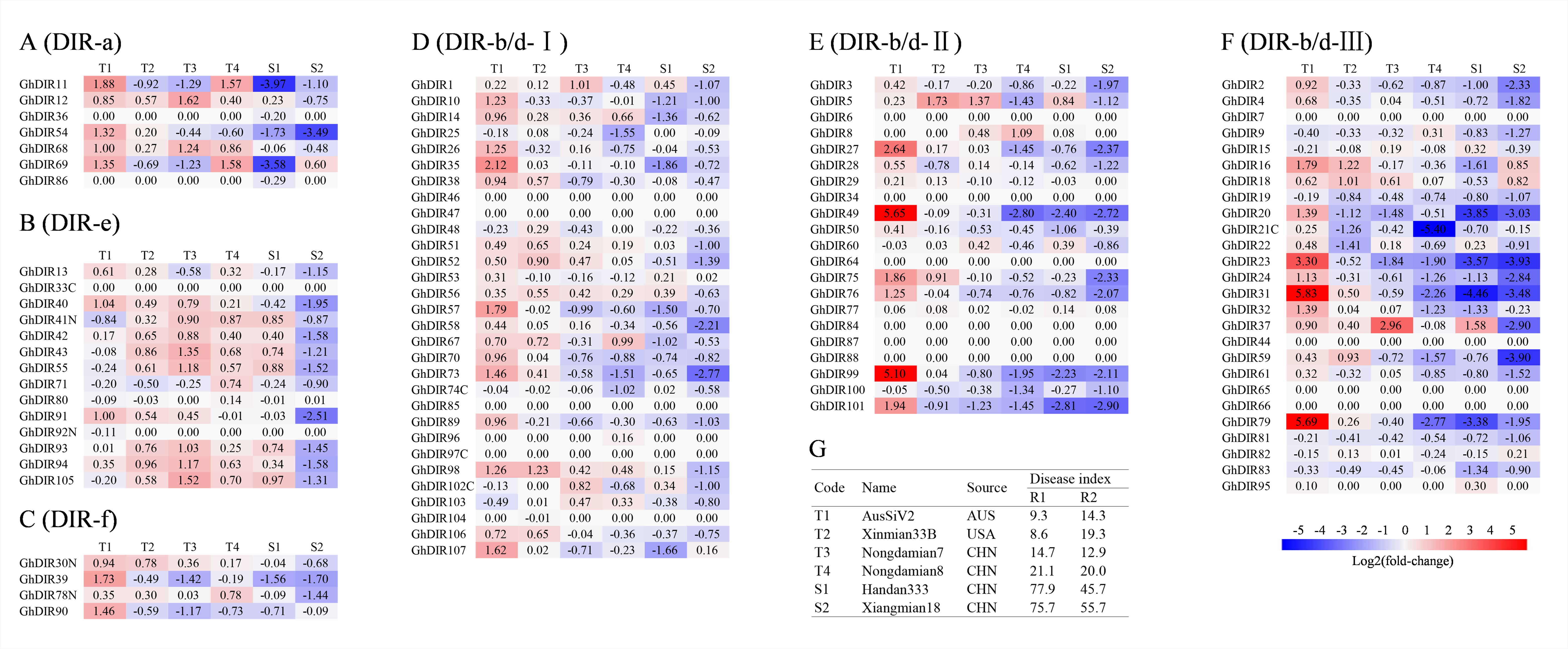

Supplement: Supplementary file 4 — Additional file 4: Figure S4. Expression patterns of GhDIRs from Verticillium wilt tolerant and susceptible cultivars (a-f). Red boxes indicate up-regulated genes after inoculation with V. dahliae (compared with the CK group), while blue boxes indicate down-regulated genes. The figures in boxes represent log2(fold-change) values corresponding to color gradients. (g) The disease index at 20 dpi. T and S represent tolerant and susceptible G. hirsutum cultivars, respectively. The original FPKM values were provided as Additional file 8: Table S12 [file 12870_2021_2859_MOESM4_ESM.tif]

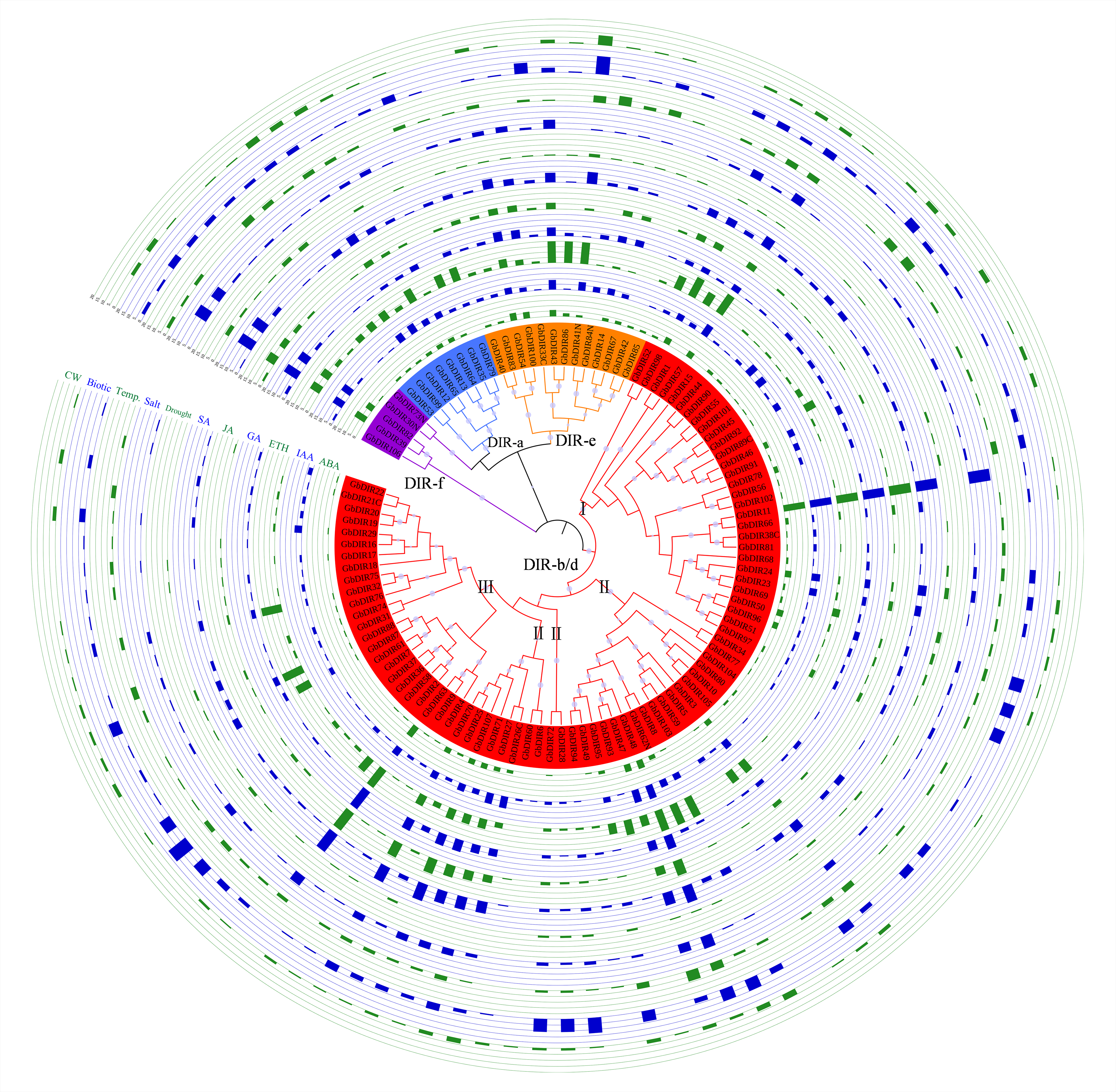

Supplement: Supplementary file 5 — Additional file 5: Figure S5. Identification of TFBS in the promoter regions of GbDIRs [file 12870_2021_2859_MOESM5_ESM.tif]

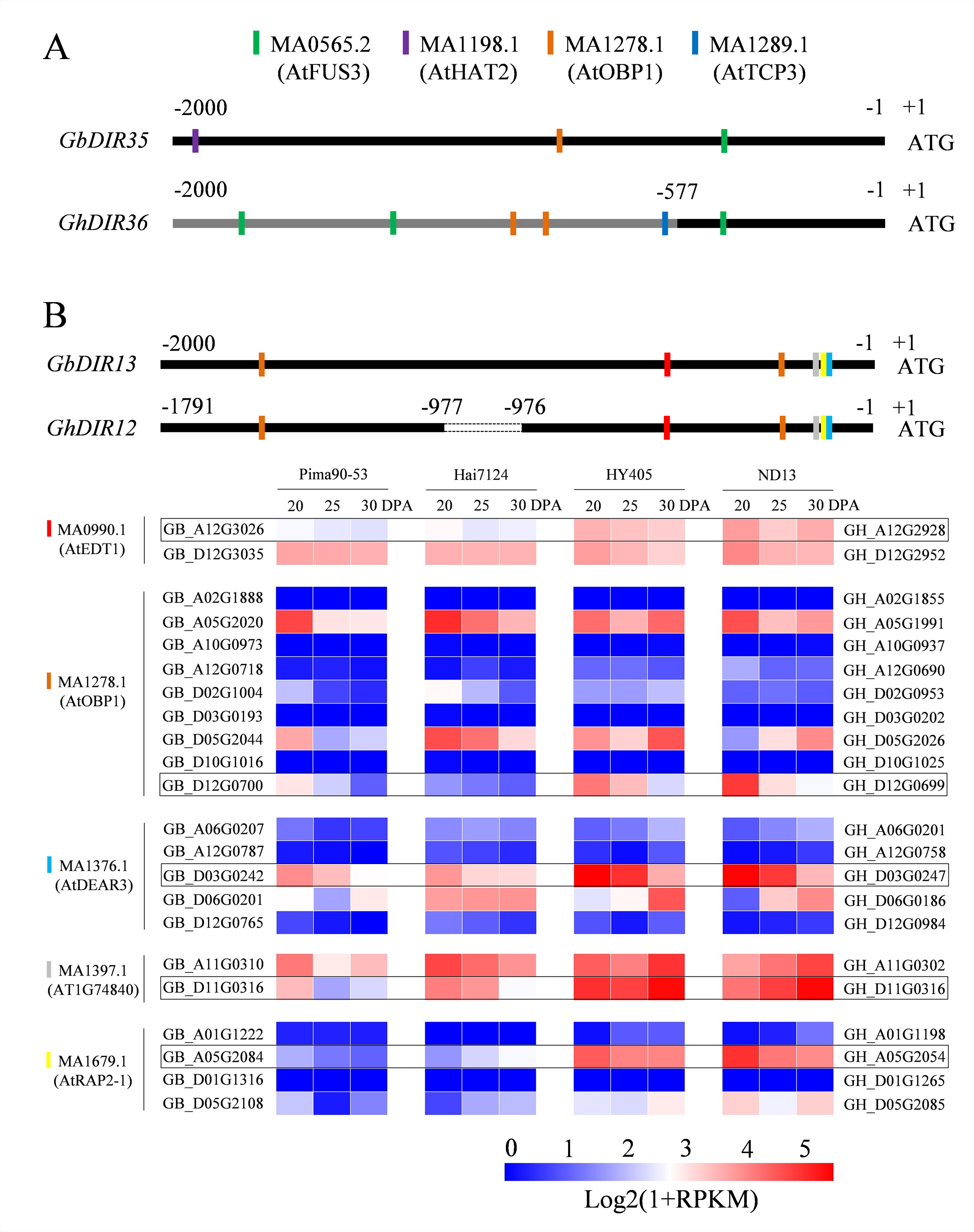

Supplement: Supplementary file 6 — Additional file 6: Figure S6. The potential mechanisms causing the differential expression. (a) GhDIR36 carried more IAA-responsive TFBS than GbDIR35. (b) Although GbDIR13 and GhDIR12 carried similar TFBS, the trans-acting TFs exhibited higher expression levels in G. hirsutum than in G. barbadense [file 12870_2021_2859_MOESM6_ESM.tif]

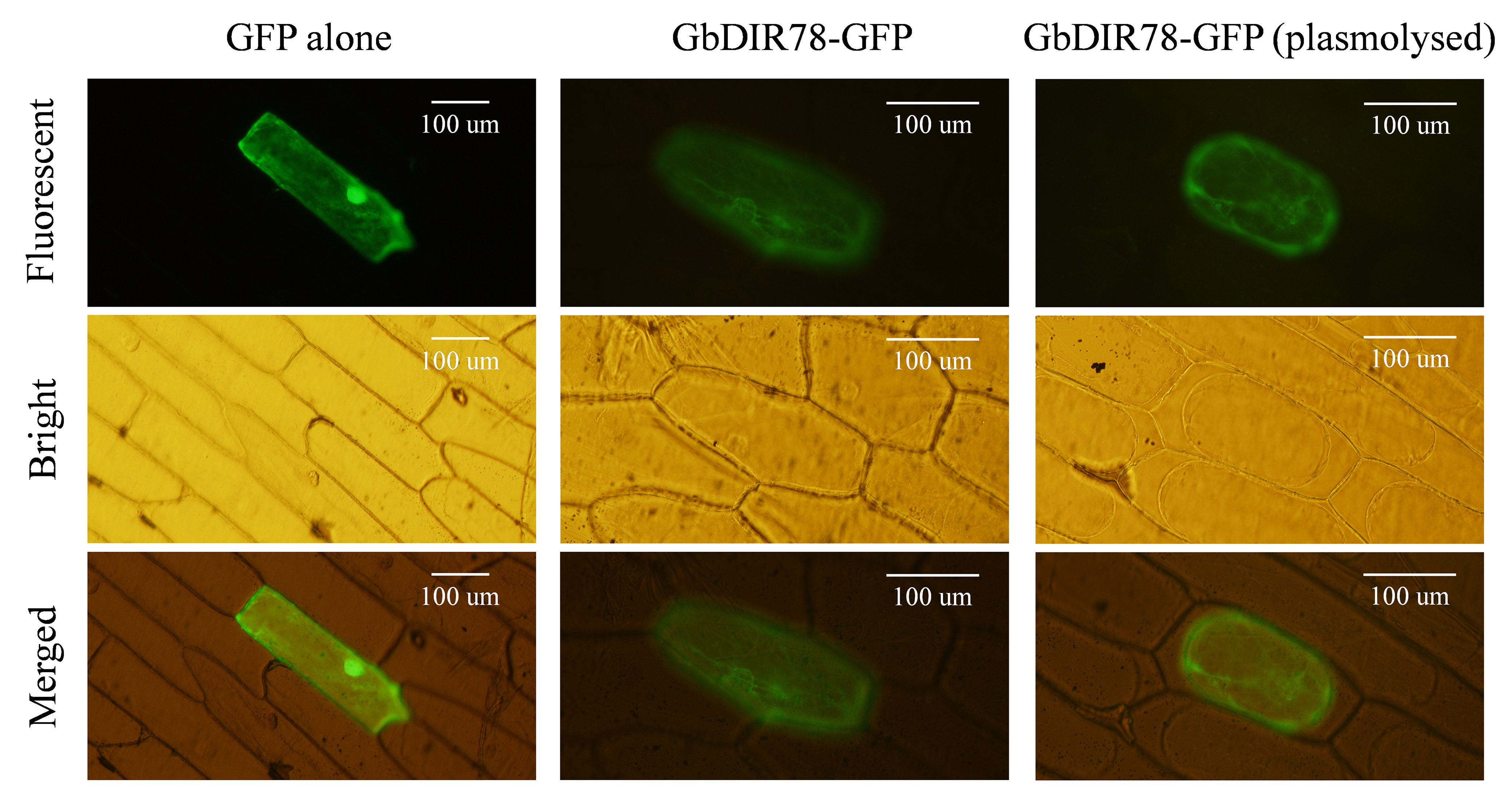

Supplement: Supplementary file 7 — Additional file 7: Figure S7. Subcellular localization of GFP alone or GbDIR78-GFP fusion protein in onion epidermal cells [file 12870_2021_2859_MOESM7_ESM.tif]
