# Supplementary material for: Evolution, expression and functional analysis of cultivated allotetraploid cotton DIR genes
Source: BMC Plant Biol. 2021 Feb 10;21:89. doi: 10.1186/s12870-021-02859-0 (PMC7876823; doi:10.1186/s12870-021-02859-0)

Western blot


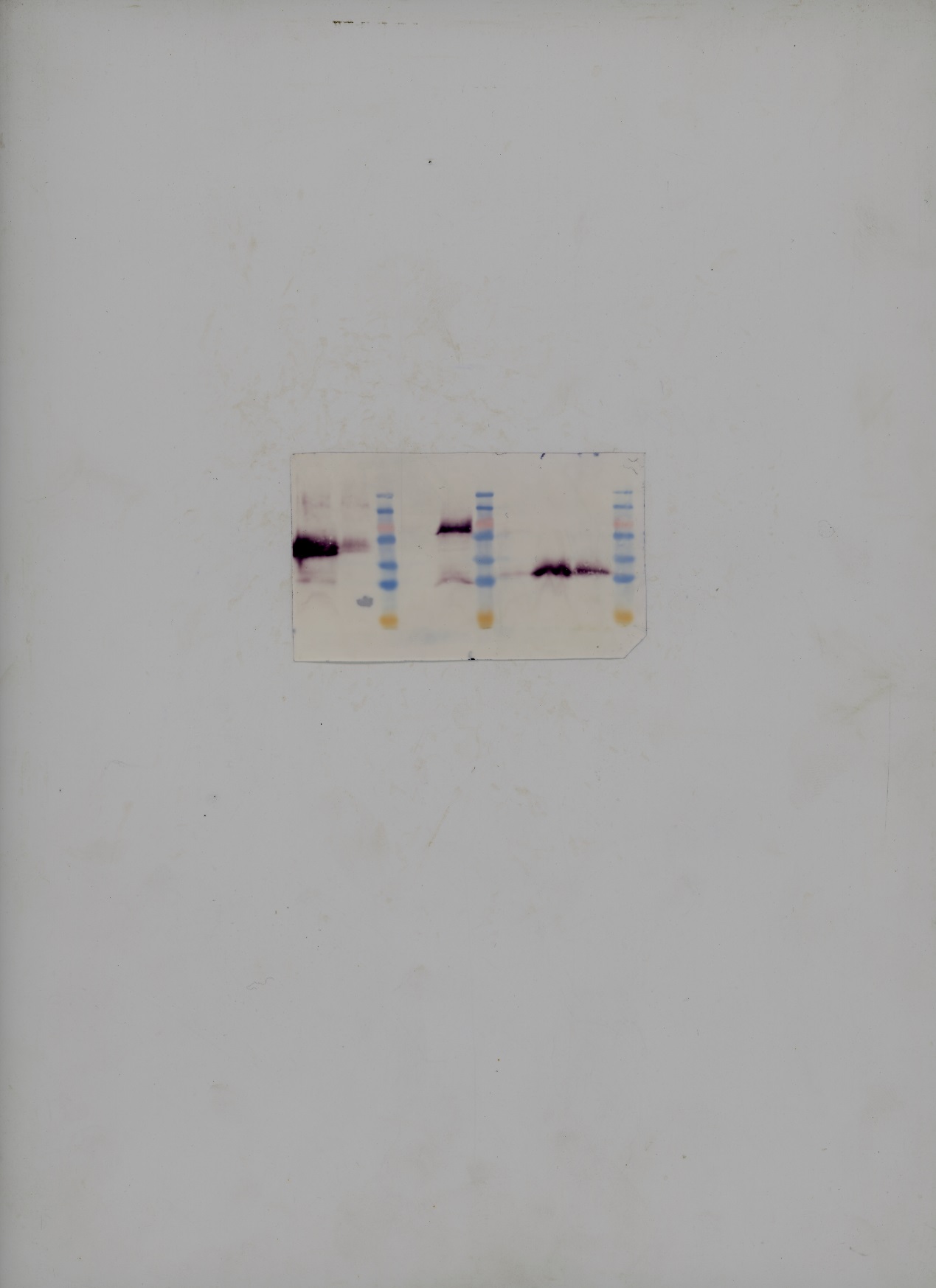


Hypocotyl epidermal cells-WT


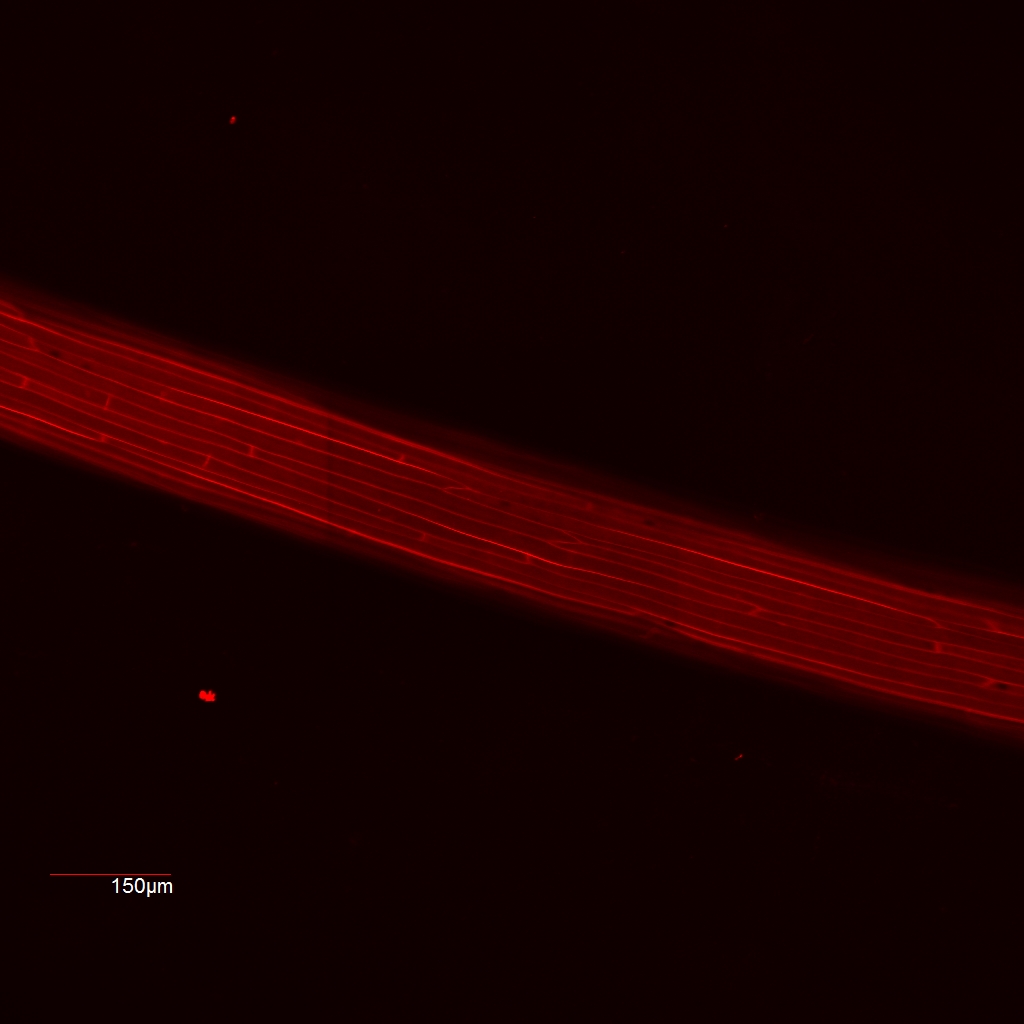


Hypocotyl epidermal cells-OE2


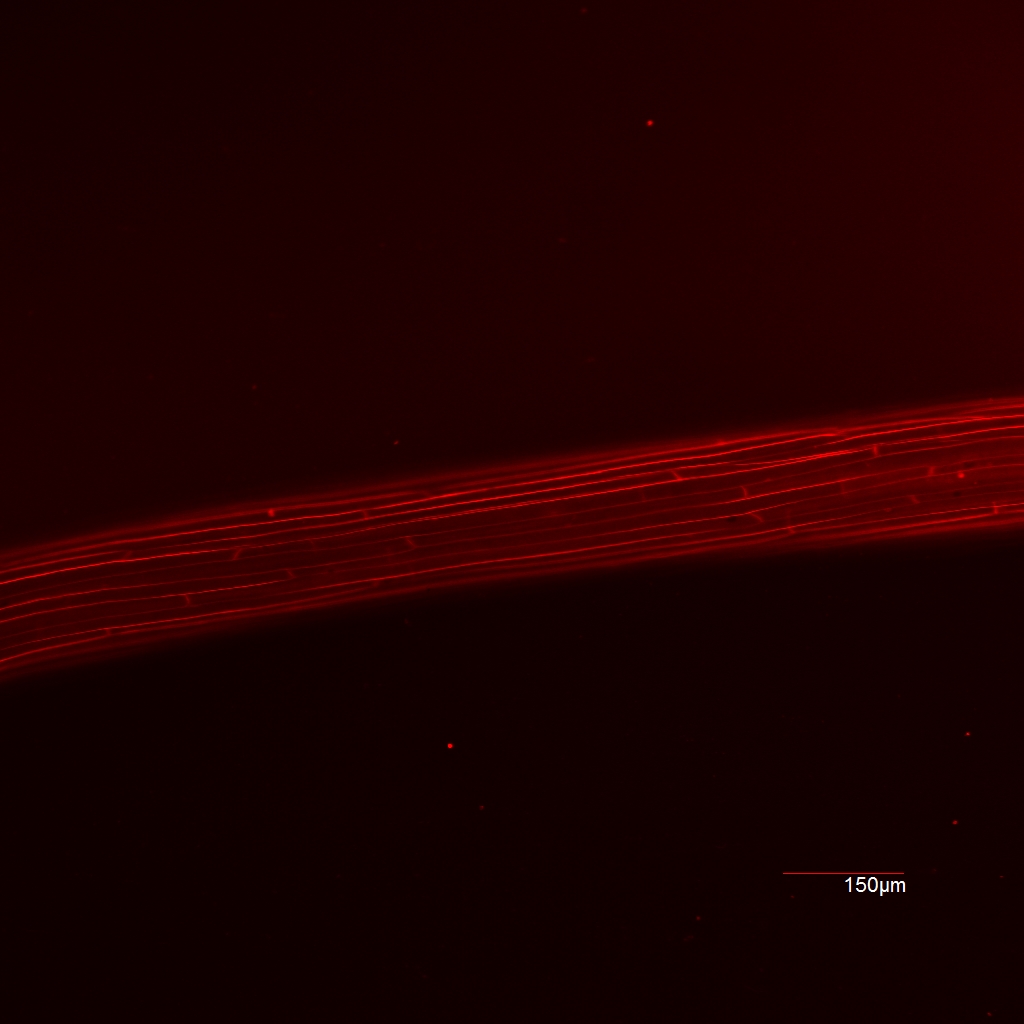


Hypocotyl epidermal cells-OE3


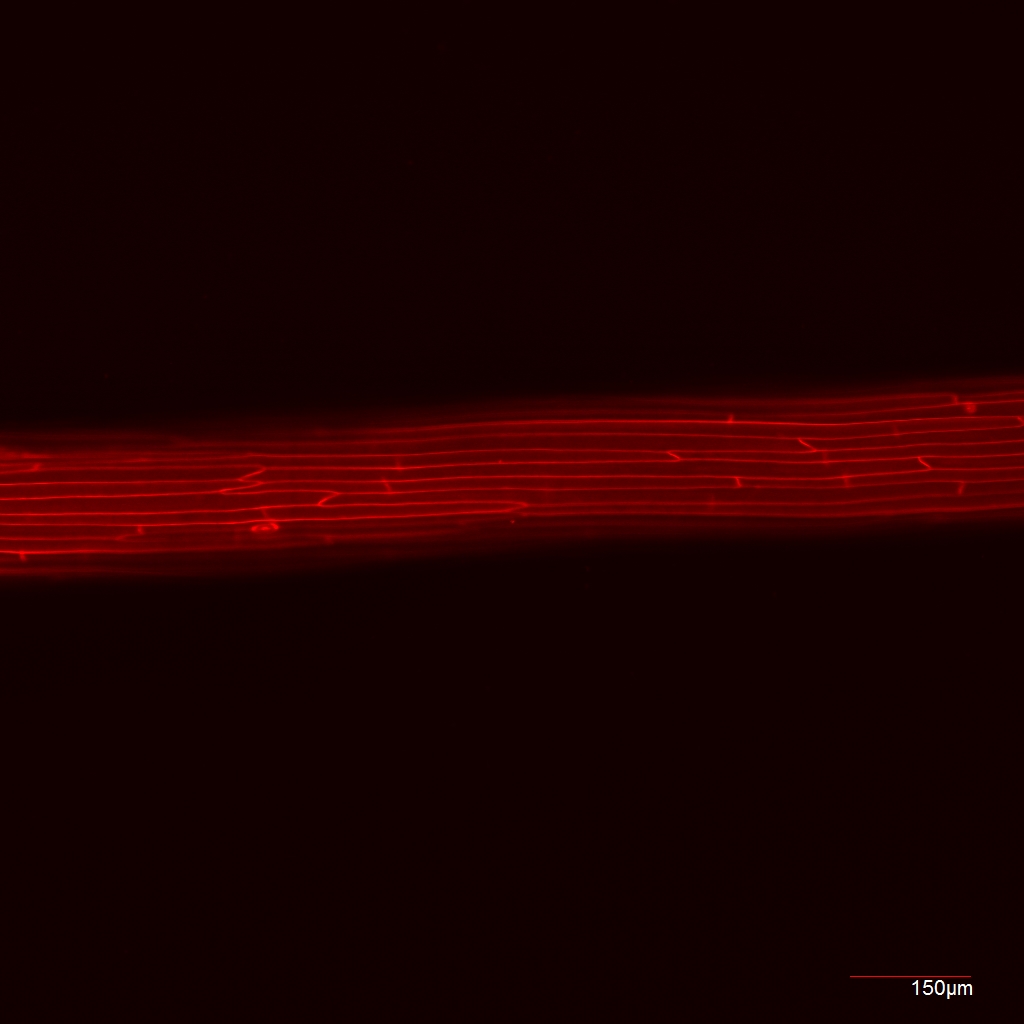


Leaf trichomes-WT


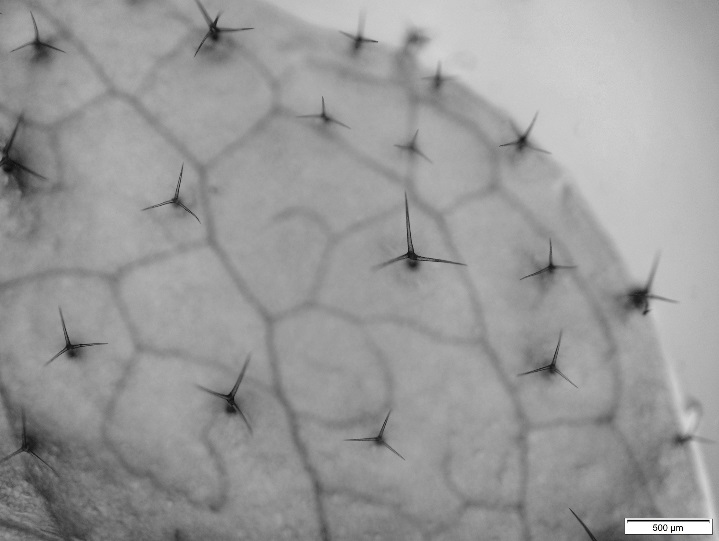


Leaf trichomes-OE


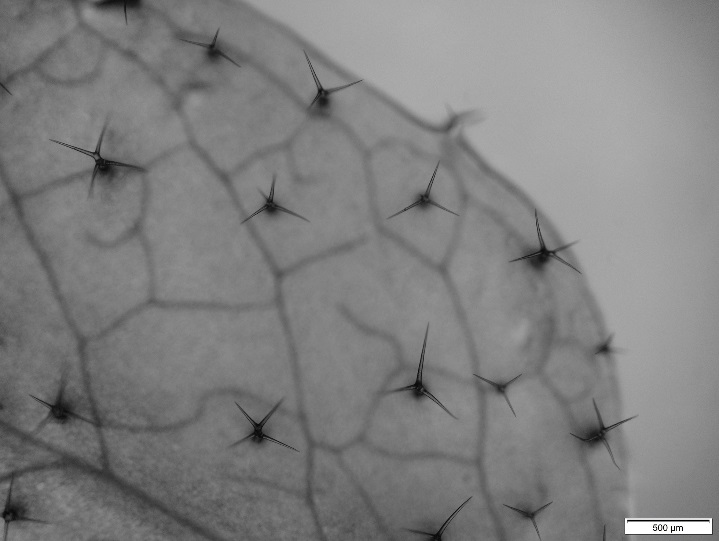

Supplement: Supplementary file 9 — Additional file 9. The original, unprocessed images [file 12870_2021_2859_MOESM9_ESM.docx]
